# Glucogallin Attenuates the LPS-Induced Signaling in Macrophages and Protects Mice against Sepsis

**DOI:** 10.3390/ijms231911254

**Published:** 2022-09-24

**Authors:** Rajveer Singh, Shivani Chandel, Arijit Ghosh, Tushar Matta, Anupam Gautam, Arka Bhattacharya, Srivalliputturu Sarath Babu, Soumi Sukla, Debasish Nag, Velayutham Ravichandiran, Syamal Roy, Dipanjan Ghosh

**Affiliations:** 1 National Institute of Pharmaceutical Education and Research, Kolkata 700054, India; 2 Netaji Subhas Chandra Bose Cancer Research Institute, 3081, Nayabad, Kolkata 700094, India; 3 Institute for Bioinformatics and Medical Informatics, University of Tübingen, Sand 14, 72076 Tübingen, Germany; 4 International Max Planck Research School “From Molecules to Organisms”, Max Planck Institute for Biology Tübingen, Max-Planck-Ring 5, 72076 Tübingen, Germany; 5 CSIR-Indian Institute of Chemical and Biology, Kolkata 700032, India

**Keywords:** beta-glucogallin, lipopolysaccharide, macrophages, gene expression profiling, anti-inflammatory, sepsis

## Abstract

The anti-oxidant and anti-inflammatory effect of beta-glucogallin (BGG), a plant-derived natural product, was evaluated in both in vitro and in vivo studies. For the in vitro study, the ability of BGG pre-treatment to quench LPS-induced effects compared to LPS alone in macrophages was investigated. It was found that BGG pre-treatment showed a significant decrease in ROS, NO, superoxide, and pro-inflammatory cytokines (TNF-alpha, IL-4, IL-17, IL-1β, and IL-6) and increased reduced glutathione coupled with the restoration of mitochondrial membrane potential. Gene profiling and further validation by qPCR showed that BGG pre-treatment downregulated the LPS-induced expression of c-Fos, Fas, MMP-9, iNOS, COX-2, MyD88, TRIF, TRAF6, TRAM, c-JUN, and NF-κB. We observed that BGG pre-treatment reduced nuclear translocation of LPS-activated NF-κB and thus reduced the subsequent expressions of NLRP3 and IL-1β, indicating the ability of BGG to inhibit inflammasome formation. Molecular docking studies showed that BGG could bind at the active site of TLR4. Finally, in the LPS-driven sepsis mouse model, we showed that pre-treatment with BGG sustained toxic shock, as evident from their 100% survival. Our study clearly showed the therapeutic potential of BGG in toxic shock syndrome.

## 1. Introduction

Natural products are well studied for their significant roles in human health benefits in relation to the prevention and treatment of inflammatory conditions and illness [1]. β-glucogallin (BGG), chemically known as 1-O-galloyl-β–d-glucopyranose, is a plant-derived polyphenolic ester and an intermediate metabolite formed in the pathway of penta-O-galloyl-glucose (β-PGG) synthesis [2,3]. The natural sources of this compound are the amla, pomegranate, raspberry, mango, Chinese white olive, etc. It has many phytopharmaceutical activities, mainly due to its anti-oxidant properties [4]. Studies show that BGG provides a protective mechanism against oxidative stress-mediated glaucoma [5]. BGG prevents LPS-induced activation of JNK and p38 and oxidative stress, thus lowering ROS levels, indicating its potent anti-inflammatory activity [6]. In Raw 267.4 macrophages, BGG attenuates the LPS-induced morphological changes and migration and inhibits activation of MMP-9 [7]. BGG is a potent and selective inhibitor of the enzyme aldose reductase (AKR1B1), which is responsible for developing oxidative stress and secondary complications in diabetes [8]. It has a significant photo-protective effect as it acts as a radical scavenger.

Exposure to extrinsic agents like bacteria, toxins, and others initiates a chemical cascade, termed an inflammatory response, to remove tissue damage and prevent the spread of infection [9,10]. However, uncontrolled inflammation propagation may lead to host tissue injuries and could prove fatal [10]. Prolonged inflammation may lead to life-threatening diseases like tuberculosis and cardiovascular diseases [11]. Initiation of inflammatory response takes place through activated macrophages, the key player of innate immunity, which increases the production of various inflammatory mediators like reactive oxygen species (ROS), nitric oxide (NO), and various inflammatory cytokines, for instance, interleukin-6 (IL-6) and tumor necrosis factor-alpha (TNF-α) [12]. A Gram-negative bacterial outer membrane component lipopolysaccharide (LPS) directly regulates various inflammatory signaling pathways in macrophages via stimulation of transcription factors like nuclear factor kappa B (NF-κB) and mitogen-activated protein kinases (MAPKs) [13]. Various pro-inflammatory markers such as cyclooxygenase-2 (COX-2), inducible nitric oxide synthase (iNOS), TNF-α, and IL-6 are produced via stimulation of monocytes/macrophages through TLR4 activation by LPS [14]. There is a proportional relationship between levels of these pro-inflammatory mediators and the severity of inflammation; thus, suppression of these mediators is key to treating inflammatory diseases [15].

NF-κB is an archetypal pro-inflammatory signaling pathway mediator which remains sequestered in the non-stimulated form [16]. Upon stimulation with LPS, phosphorylation and ubiquitylation of this protein occur, promoting the translocation of its active form into the nucleus, which acts as transcription factors for many pro-inflammatory cytokines genes [17]. Direct inhibition of NF-κB translocation to the nucleus can proficiently lessen the expression of pro-inflammatory cytokines, iNOS, and COX-2. Pathogens are detected on the surfaces of macrophages via TLRs, which belong to the pattern recognition receptors (PRRs) class that initiate innate immune responses [18]. The most abundant and pivotal TLR4 complex is responsible for recognizing LPS which, upon attachment with TLR4, recruits adaptor proteins like myeloid differentiation protein 2 and LPS binding protein. This further activates nuclear factor-κB (NF-κB) and MAPKs, which ultimately induce pro-inflammatory gene expression [19]. Uncontrolled gene expression of pro-inflammatory markers could result in myriad inflammatory diseases like autoimmune and neurodegenerative conditions, cancer, non-alcoholic fatty liver disease, and cardiovascular diseases [20].

Along with its role in inflammation, LPS is also a potent apoptotic agent [21]. Apoptotic activity of LPS may vary among different cell lines due to differences in expression levels of the same genes in different cells. LPS-induced apoptosis and pro-inflammatory events are of greater clinical relevance associated with endotoxemia [22]. In our recent study, we found that, BGG has protective effect against Arsenic trioxide induced toxicity in RAW 264.7 [23]. 

In the present study, we have investigated the effects of BGG in attenuating the LPS-induced activation signaling in macrophages (both in RAW 264.7 and mice peritoneal) and a mouse sepsis model induced by intraperitoneal injection of LPS. A detailed study was undertaken to characterize the gene expression profiling and unravel the detailed signaling pathway to decipher the mechanism of action involved. Our results show that BGG decreased the expression of pro-inflammatory cytokines and inhibited the activation of the TLR4/NF-κB and NLRP3 pathways. BGG also protected LPS-induced sepsis mice and reversed the pro-inflammatory cytokine production and organ toxicity.

## 2. Results

### 2.1. Cell Cytotoxicity Assay

BGG is a gallo-tannin; the chemical structure is shown in Figure S1A.

Before investigating the anti-inflammatory effect of BGG against LPS-induced inflammation, we first checked the in vitro cytotoxicity effect of BGG (1 μM to 100 μM) in RAW 264.7 cells and peritoneal macrophages; at up to 48 h, BGG showed no cytotoxicity as determined by MTT assay (Figure S1B–E). We also analyzed the effect of BGG on LPS-induced cytotoxicity in RAW 264.7 cells at 24 and 48 h through an MTT assay, which resulted in no significant difference found at 24 h of treatment. But a decrease in cell viability was observed at the end of 48 h; the cell viability in the pre-treated BGG followed by treated LPS increased the cell viability, which was almost similar to that of the control cells (Figure S1F–I).

### 2.2. Effect of BGG on ROS Production in the Macrophage with and without LPS Induction

The production of reactive oxygen species is one of the significant causes of inflammation-mediated toxicity. To determine the effect of BGG on ROS production, we have treated the RAW 264.7 cells with different concentrations (1 μM to 100 μM) of BGG. After 24 h, ROS assay was done by flow-cytometric analysis after staining the cells with ROS-sensitive fluorophore 2′,7′-dichlorofluorescein diacetate (DCFDA) dye. Results showed no significant changes compared to the control (Figure S2A,B).

**Figure 1:**
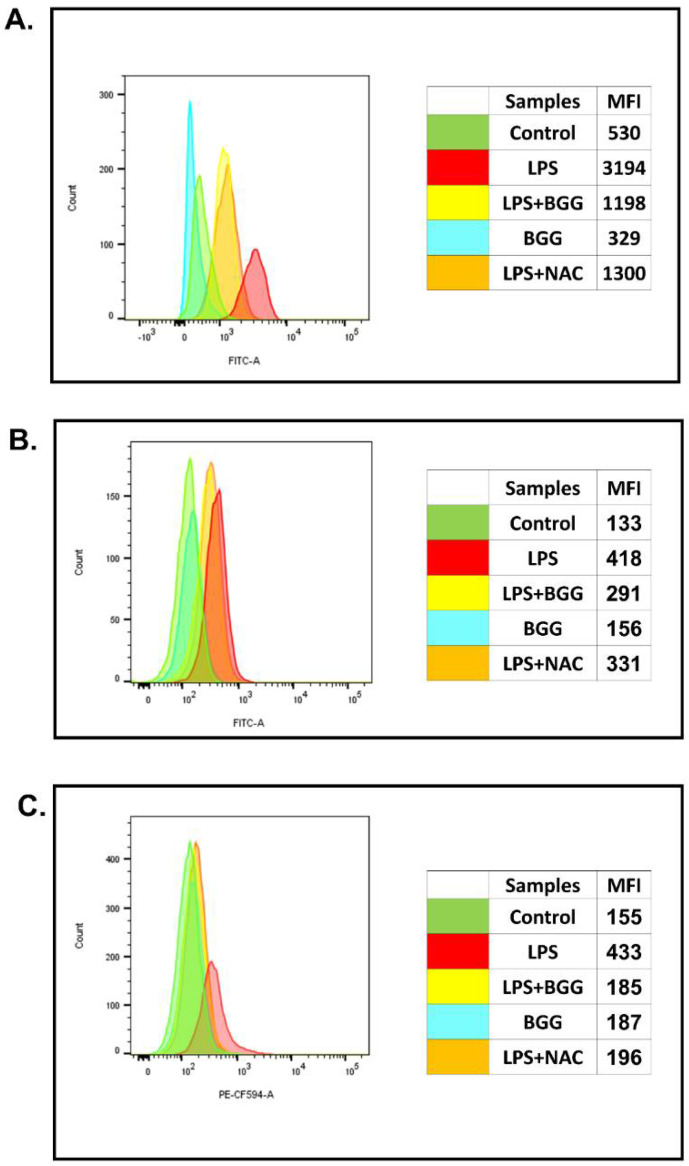
RAW 264.7 cells and mice peritoneal macrophages were pre-treated with BGG (10 μM) and NAC (10 μM) for 1 h, followed by LPS (1 μg/mL) stimulation for 23 h. BGG attenuates the LPS-induced ROS production in (**A**) RAW 264.7 and (**B**) in mice peritoneal macrophages, and (**C**) BGG protects LPS-induced superoxide production in RAW 264.7 macrophages, measured by MitoSox Red. Results are mentioned in mean fluorescence intensity.

We also checked whether pre-treatment with BGG could reduce the LPS-induced ROS production in macrophage cells (in both RAW 264.7 cell line and peritoneal macrophage). Pre-treatment with BGG (10 μM), followed by induction with LPS (1 μg/mL), showed a significant reduction in ROS levels both in RAW 264.7 cells and peritoneal macrophage cells, as indicated by MFI values (Figure 1A,B). We used N-acetylcysteine (NAC), a known ROS scavenger, as a positive control. In RAW 264.7 cells and peritoneal macrophages, the treatments with LPS alone increased MFI values to 3194 (control value 530) and 418 (control value 133), respectively, whereas pre-treatment with BGG reduced these values to 1198 and 298, respectively.

This result suggests the protective activity of BGG against LPS-induced ROS production in macrophages, and the antioxidative activity of BGG is similar to that of NAC.

To analyze the superoxide production in the mitochondria of live cells, the RAW 264.7 cells were pre-treated with BGG (10 μM) for 1 h, followed by LPS (1 μg/mL) for 23 h, which showed that BGG pre-treatment successfully reduced the LPS-induced superoxide production (Figure 1C).

### 2.3. Attenuation of NO Production by BGG in the LPS-Induced Macrophages

To evaluate the effect of BGG-induced nitric oxide generation in the macrophages, the RAW 264.7 cells were treated with different concentrations of BGG (1 μM to 100 μΜ) for 24 h. There was no significant change in NO generation by BGG compared to the control (Figure S2C).

**Figure 2:**
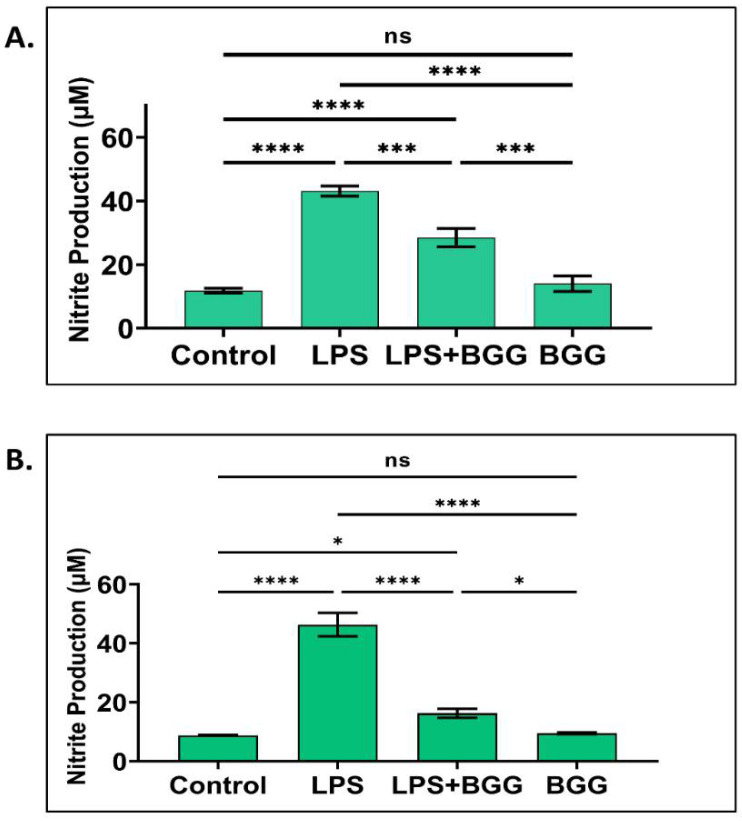
BGG attenuates the LPS-induced NO production in (**A**) RAW 264.7 and (**B**) mice peritoneal macrophages. Results are mentioned as mean ± SD (*n* = 5), *p*-value ≤ 0.05. (* *p* ≤ 0.05, *** *p* < 0.001, and **** *p* < 0.0001) (ns: not significant).

We further checked the protective effect of BGG on LPS-induced NO production in RAW 264.7 cells and mice peritoneal macrophages. Macrophages were pre-treated with BGG (10 μM) for 1 h, followed by treatment with LPS (1 μg/mL) for 23 h. The BGG pre-treatment significantly reduced LPS-induced NO production in RAW 264.7 (43.13 ± 1.6 µM to 28.48 ± 2.9 µM) and mice peritoneal macrophages from (45.04 ± 4.6 µM to 16.27 ± 1.5 µM) (Figure 2A,B).

### 2.4. BGG Attenuates Mitochondrial Membrane Potential—Damaged by LPS

**Figure 3:**
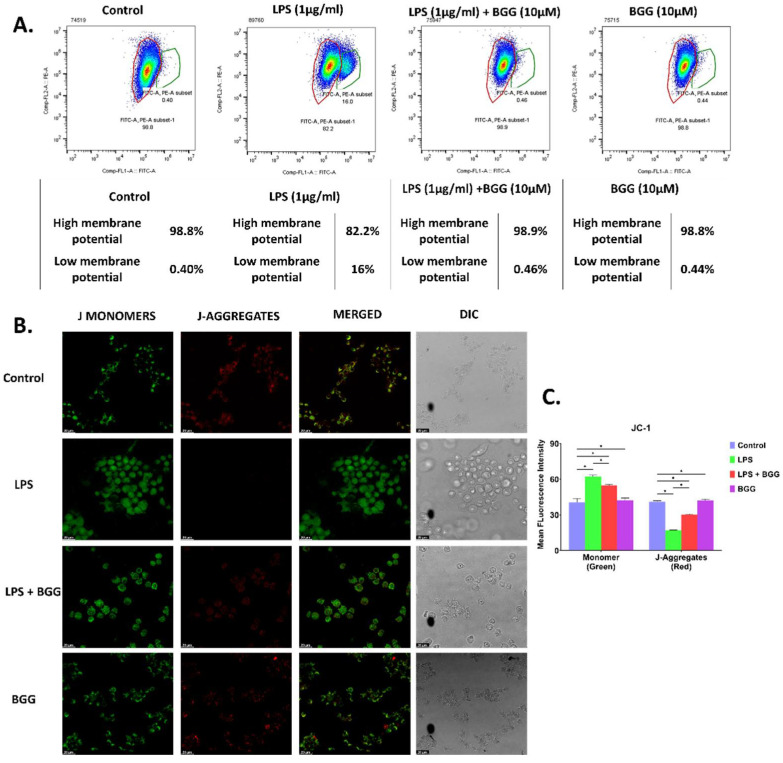
BGG attenuates the LPS-induced mitochondria membrane potential depolarization. RAW 264.7 cells were pre-treated with BGG (10 μM) for 1 h, followed by LPS (1 μg/mL) for 23 h of stimulation. (**A**) BGG attenuates the LPS depolarized membrane potential measured by flow cytometry in RAW 264.7 cells. (**B**) JC-1 staining showing BGG attenuates the LPS depolarized membrane potential measured by confocal microscopy in RAW 264.7 cells. (**C**) The graph represents the mean fluorescence intensity showing the J-monomer and J-aggregates measured by confocal microscopy (Mean ± SEM) (*n* = 3), *p*-value ≤ 0.05. (* *p* ≤ 0.05).

The maintenance of mitochondrial transmembrane potential (ΔΨm) is a critical process for the generation of ATP throughout the life of a cell. Oxidative stress due to the accumulation of ROS can cause a significant loss in ΔΨm, which may lead to the death of cells due to the depletion in energy generation. To determine the effect of BGG on the maintenance of ΔΨm, we treated cells with only LPS, pre-treated BGG along with LPS treatment, and only BGG for 24 h. Then cells were stained with a dual-emission potential dye JC-1 (5,5′,6,6′-tetrachloro-1,1′,3,3′-tetraethyl-imidacarbocyanine iodide) which remains as a monomer at low ΔΨm emitting green fluorescence, whereas in standard or high ΔΨm it forms red fluorescent J-aggregates. Flow-cytometric analysis showed only red fluorescence in cells with no treatment. Nevertheless, the cells exposed to LPS showed a reduction in red fluorescence and a shift toward green fluorescence in about 16% of the total population. Pre-treatment with BGG on LPS-treated cells showed retention of red fluorescence similar to control and only BGG-treated cells (Figure 3A). We further confirmed the data by using confocal microscopy, and we saw a significant change in the fluorescent pattern from punctate red fluorescence to a diffuse green fluorescence only in LPS-treated cells compared to control, indicating a disruption of ΔΨm. BGG pre-treated, LPS-treated cells showed a significant reduction in green fluorescence compared to the solely LPS-treated cells, indicating the attenuation of mitochondrial membrane potential by BGG. However, the only BGG-treated cells showed no significant difference in red and green fluorescence patterns compared to the control (Figure 3B,C).

Both confocal and flowcytometric data confirmed that BGG pre-treatment significantly reversed the LPS-disrupted mitochondria membrane potential by incrementing red aggregates and decreasing green monomers.

### 2.5. Effect of BGG on LPS-Stimulated Gene Expression in RAW 264.7 Macrophages Cells

RAW 264.7 cells were pre-treated for 1 h with BGG (10 µM), and then LPS (1 μg/mL) was added to the incubation medium for another 23 h. The control treatment with BGG alone lasted 24 h and LPS alone for 23 h. RNA was isolated, prepared from the four samples (in duplicate), and analyzed to assess the gene expression transcriptional expression level changes. The raw data of the samples were imported from text files by using R packages limma. Probes with poor signals were removed by using the signal distribution from negative control probes. The normalization of the samples was done with the R package limma using the quantile method.

**Figure 4:**
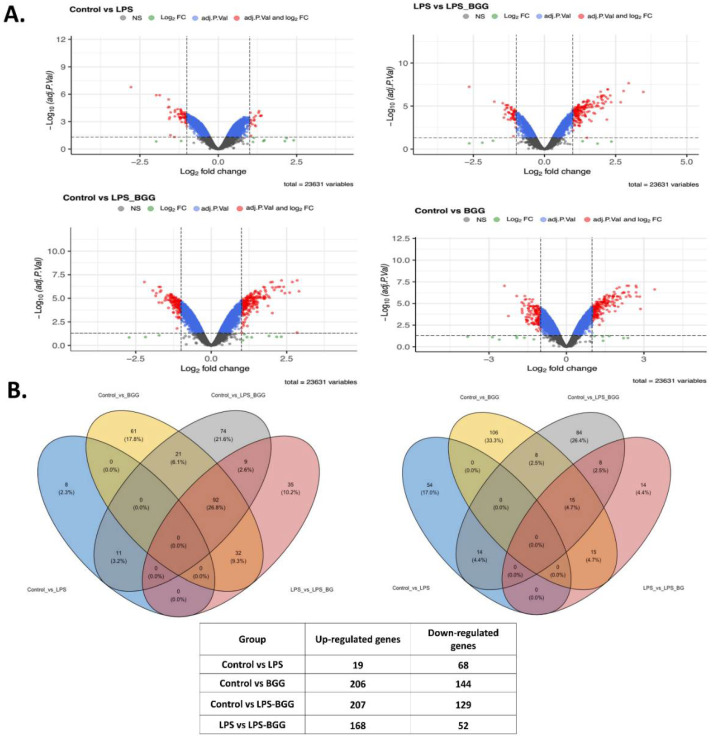
Microarray-based gene expression profiling on RAW 264.7 macrophages. (**A**) Volcano plots, represents the total number of genes found in each group (control vs. LPS, LPS vs. LPS-BGG, control vs. LPS-BGG, and control vs. BGG). (**B**) Wind diagram represents the differential expressed genes found in four groups, including the upregulated and downregulated gene.

In gene expression, a total of 23,631 genes were found in the four groups, as shown by the volcano plot each group presents on the x-axis. Values with adjusted *p* value less than 0.05 and log^2^ fold change more remarkable than 1 or less than −1 were considered significant (color in red) (Figure 4A). Further estimation of important differential features was utilizing the R packages limma. Enrichment of functional annotation was performed with R package gprofiler2. Significant differential features were obtained by using thresholds adj. *p* value ≤ 0.05 and abs(logFC) ≥ 1, revealing that 220 genes were differentially expressed in the LPS vs. LPS-BGG group.

**Figure 5:**
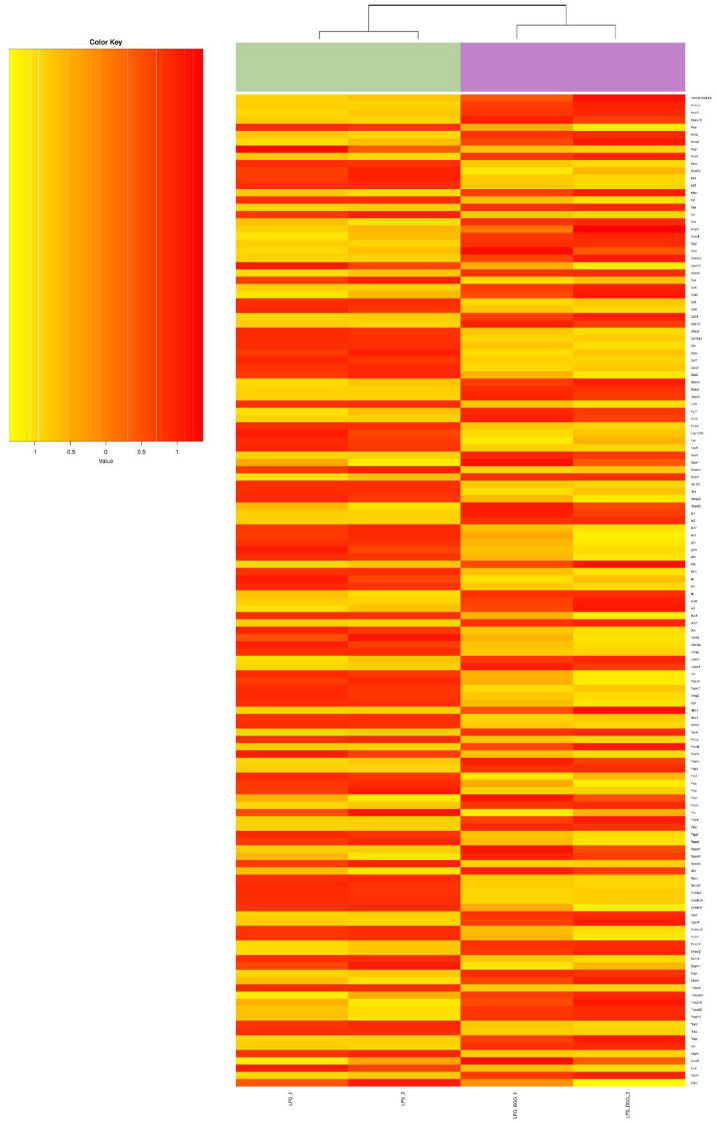
Heatmap represents the genes involved in the cell death pathway from microarray gene expression.

**Figure 6:**
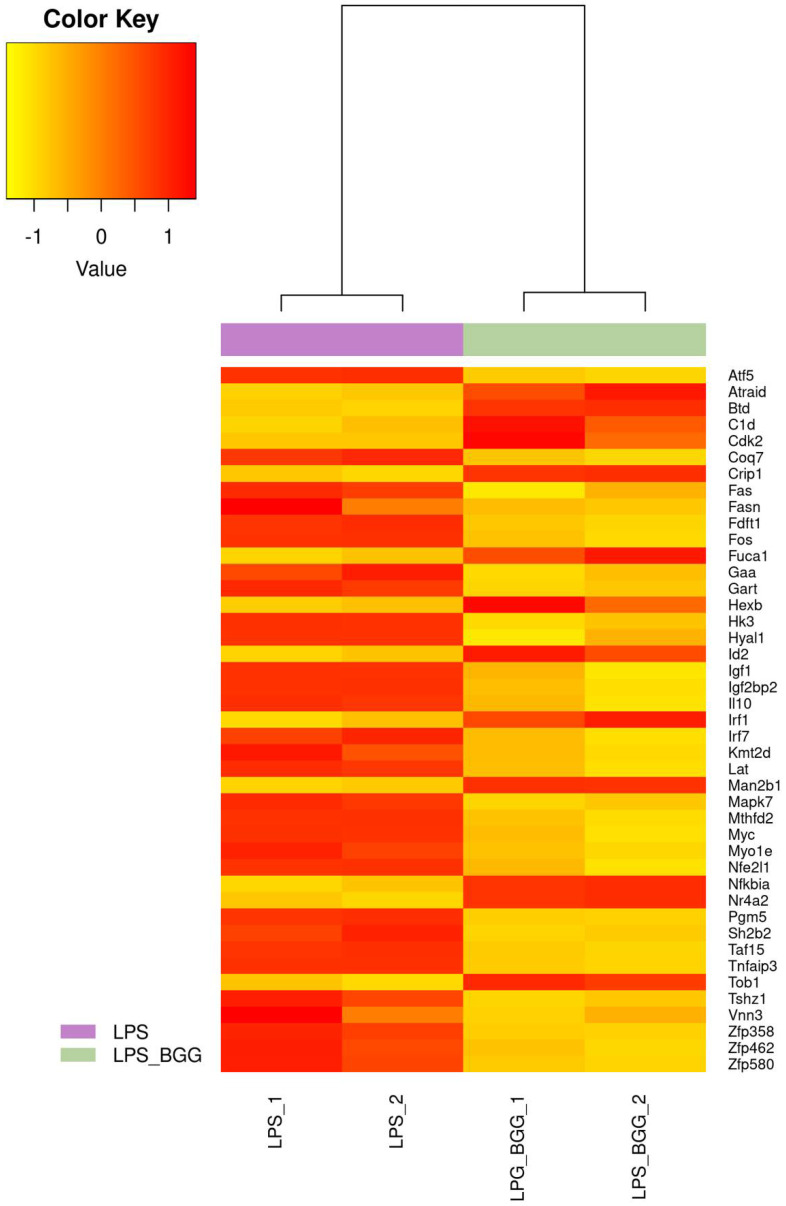
Heatmap represents the genes involved in the metabolic system pathway from microarray gene expression.

**Figure 7:**
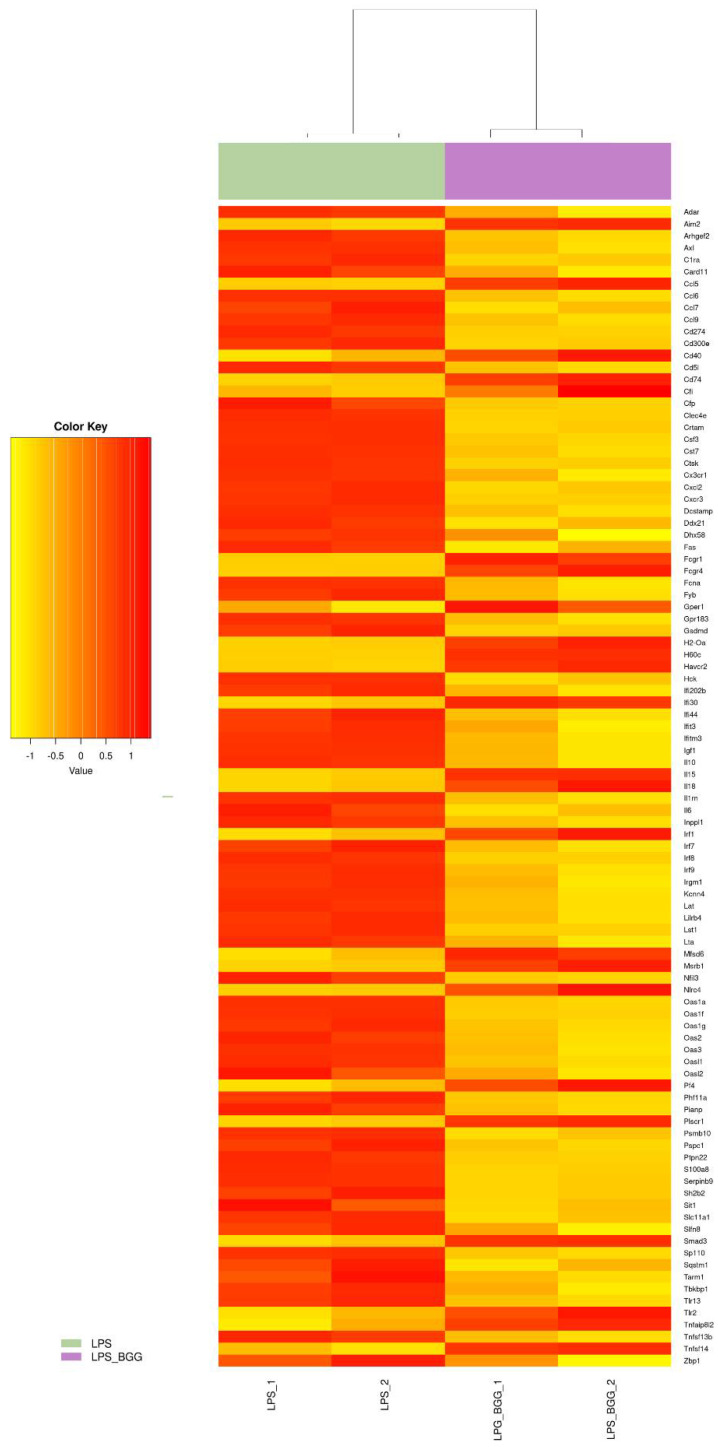
Heatmap represents the genes involved in the immune system pathway from microarray gene expression.

Of these, 68 genes were downregulated after treatment with LPS; and from these numbers of genes, 19 transcripts were found to be upregulated. The BGG + LPS treatment was analyzed, 207 genes were upregulated, and BGG + LPS downregulated 129 genes. After treatment with BGG, 206 transcripts were upregulated, and 144 were downregulated. From these genes, 168 gene products were upregulated and 52 downregulated exclusively due to the combination of BGG + LPS and were not affected by the LPS group (Figure 4B). In order to annotate the functional relationship between these groups of LPS-upregulated genes and downregulated genes, gene ontology (GO) analysis was conducted by using the DAVID bioinformatics resources and KEGG pathways. Most LPS-upregulated transcripts involved all major biological processes, cellular components, and molecular functions in cell death, metabolic process, and immune system pathway (Figure 5, Figure 6 and Figure 7).

Genes associated with toll-like receptor (TLR) pathways, antigen processing, and MAPK pathways were included in the analysis of the KEGG database. DAVID represented genes within specific GO categories in IPA global functional analyses of upregulated and downregulated genes (≥1-fold; *p* ≤ 0.005) by LPS. According to IPA analysis of the upregulated LPS transcripts, cell-to-cell signaling, cell movements, growth and proliferation, immune response, and signaling are among the significant functions of these genes; canonical and non-canonical NF-κB pathway genes also were studied.

### 2.6. Validation of Microarray Results by qPCR

We have identified important genes mainly linked with the TLR-4 pathways and validated them with qPCR.

**Figure 8:**
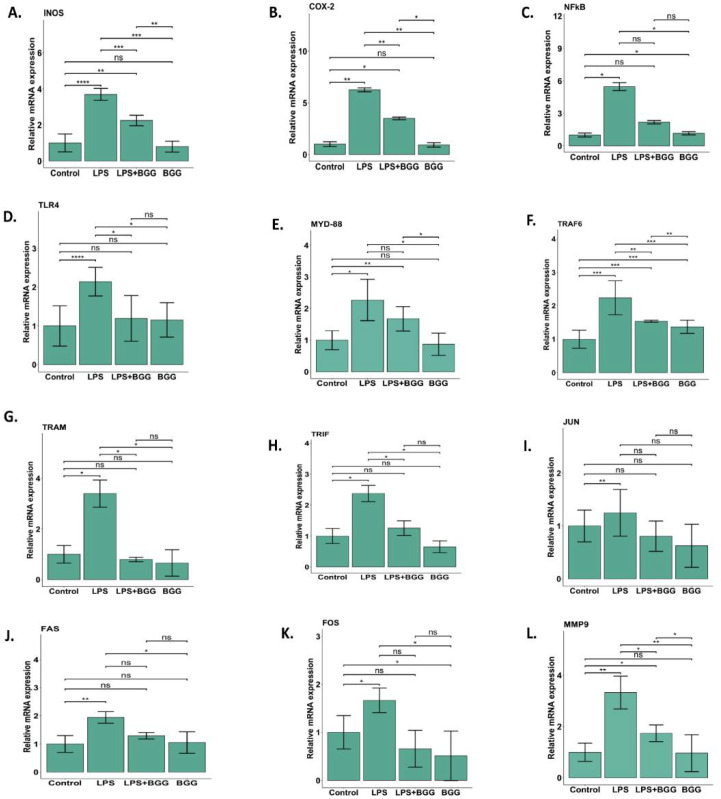
Validation of the gene profiling data, RAW 264.7 cells were pre-treated with BGG (10 μM) for 1 h, followed by LPS (1 μg/mL) stimulation for 23 h. Total RNA was isolated by using TRIzol reagent; the isolated RNA was subjected to cDNA synthesis, and SYBERgreen was used for amplification. The qPCR confirmed cell death, immune system, and metabolic process pathway genes. Actin was used as a housekeeping gene. Results are mentioned as mean ± SD (*n* = 3), *p*-value ≤ 0.05, (* *p* ≤ 0.05, ** *p* < 0.001, *** *p* < 0.0001, and **** *p* < 0.00001). (**A**–**L**) (ns: not significant).

The qPCR was repeated in triplicates by using three mRNA preparation with independent experiments. The results found from the qPCR were measured as relative fold changes compared to the control experiment. In almost all cases, there has been an excellent agreement between the microarray and the qPCR data in terms of both the direction and extent of change. The qPCR data showed that LPS significantly upregulates the expression of iNOS (Figure 8A), COX-2 (Figure 8B), NF-κB (Figure 8C), TLR4 (Figure 8D), MYD-88 (Figure 8E), TRAF6 (Figure 8F), TRAM (Figure 8G), TRIF (Figure 8H), c-JUN (Figure 8I), FAS (Figure 8J), c-FOS (Figure 8K), and MMP-9 (Figure 8L) genes, which further downregulated in the pre-treatment of the BGG.

### 2.7. BGG Attenuates LPS-Induced Pro-Inflammatory Cytokines Productions and Inflammation in Macrophages Cells

#### 2.7.1. Measurement of Extracellular Cytokines by Cytometric Bead Array (CBA)

**Figure 9:**
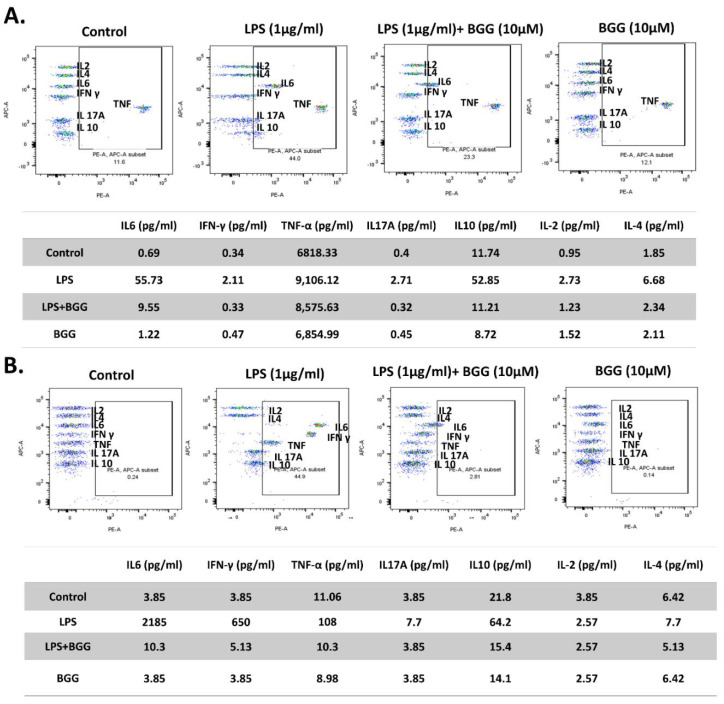
Measuring extracellular cytokine interleukins by CBA bead assay analyzed by using flow cytometry in (**A**) RAW 264.7 and (**B**) mice peritoneal macrophages.

A cytometric bead array (CBA) was done to check the change in the cytokines excretion by the macrophages, cultured in vitro for simultaneous measurement of multiple cytokines in small volumes more precisely than the traditional immunoassays. After 24 h of LPS treatment in the RAW 264.7 and mice peritoneal macrophages cell line, interleukins IL-6, IFN-γ, TNFs, IL-17A, IL-10, IL-2, and IL-4 showed a significant increase in concentration compared to control cells. The BGG pre-treatment decreased IL-6, IFN-γ, IL-10, and IL-7A excretion by around 5- to 6-fold and IL-2 and IL-4 excretion by approximately 2- to 3-fold compared to LPS treated RAW 264.7 cells. Concentrations of TNF, IL-6 and IL-10 decreased from 9106.12 pg/mL, 55.73 pg/mL and 52.85 pg/mL in LPS-treated RAW 264.7 cells to 8575.63 pg/mL, 9.55 pg/mL and 11.21 pg/mL, respectively, when pre-treated with BGG (Figure 9A). Similarly, in the case of mice peritoneal macrophages, BGG pre-treatment followed by LPS treatment led to a significant reduction in extracellular IL-6, IFN-γ, TNFs, IL-17, and 1L-10 levels (Figure 9B). Only BGG treatment showed excreted concentration of interleukins similar to control macrophages. 

#### 2.7.2. Intracellular Staining of Interleukins

We have performed the flow cytometric analysis for intracellular expression of interleukins TNFs, IFN, and IL-10 in the RAW 264.7 cell. After 24 h of treatment, LPS-treated cells showed a significant surge in the percentage of cells expressing the interleukin TNFs, IFN, and IL-10 compared to control cells. The BGG prior treatment (1 h) with LPS-treated cells showed a decrease in the percentage of interleukin-producing cells compared to only LPS-treated cells. At the same time, only BGG treatment showed no significant change in expression compared to the untreated cells (Figure S3A–D).

### 2.8. BGG Attenuates the LPS-Induced Inflammation and Pyroptosis in RAW 264.7 Macrophages via NF-κB/NLRP3 Pathway

**Figure 10:**
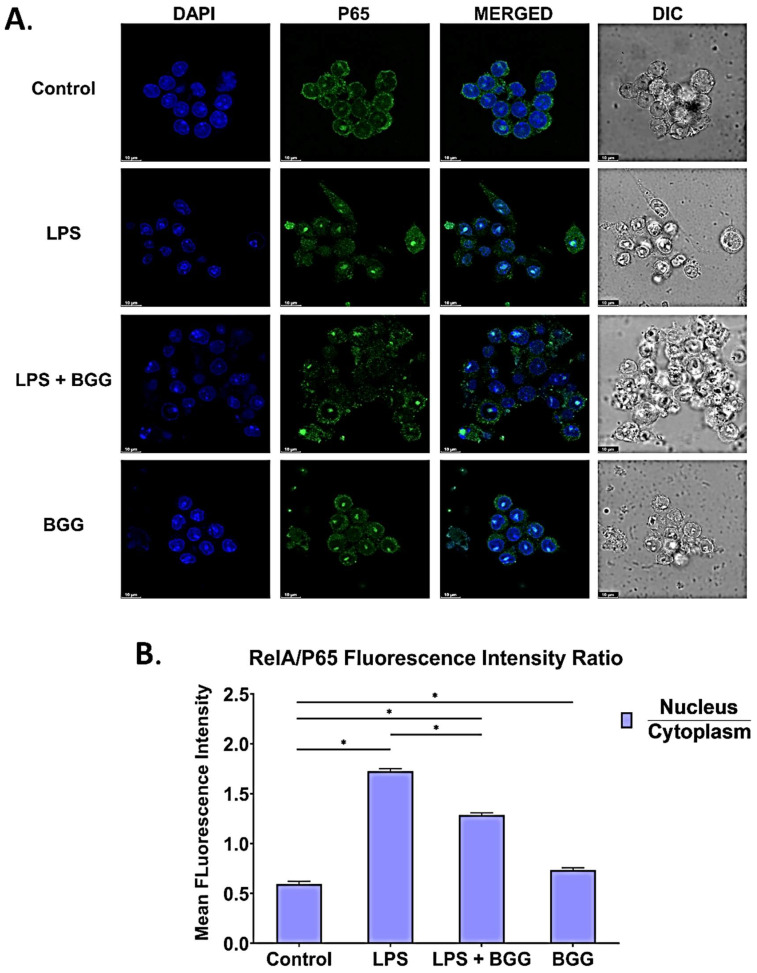
Immunofluorescence staining images representing the BGG attenuation of the LPS-enhanced translocation of p65 from the cytosol to the nucleus measured by confocal microscopy. (**A**) Represents the translocation assay of p65 in RAW 264.7 macrophages. (**B**) Represents the mean fluorescence intensity showing the p65 ratio of nucleus vs. cytoplasm. Results are mentioned as mean ± SD (*n* = 3), *p*-value ≤ 0.05, (* *p* ≤ 0.05).

**Figure 11:**
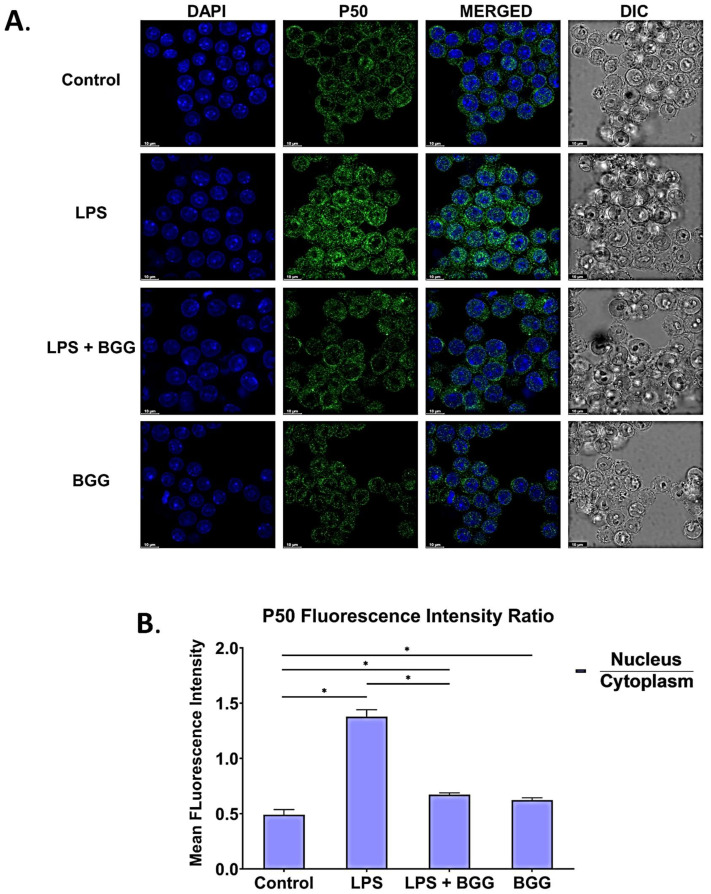
Immunofluorescence staining images representing the BGG attenuation of the LPS-enhanced translocation of p50 from the cytosol to the nucleus measured by confocal microscopy. (**A**) The translocation assay of p50 in RAW 264.7 macrophages. (**B**) The mean fluorescence intensity showing the p50 ratio of nucleus vs. cytoplasm. Results are mentioned as mean ± SEM (*n* = 3 independent experiments) (* *p* ≤ 0.05). Scale bars: 10 µm.

NF-κB has a vital role in inflammation. To analyze the effects of BGG on NF-κB activation in LPS-treated RAW 264.7 cells, translocation was measured by p65 and p50 with immunofluorescence staining. Figure 7 showed that LPS enhanced the translocation of p65 and p50 from the cytoplasm to the nucleus, activating inflammatory signals in the canonical pathway. The pre-treatment with BGG suppressed the translocation of p65 and p50 from the cytoplasm to the nucleus (Figure 10 and Figure 11). We also measured the translocation of RelB by immunofluorescence staining on LPS-stimulated RAW 264.7 cells to assess the effect of BGG on the non-canonical pathway. We found no effect of LPS and BGG on RelB levels (Figure S4A,B). Hence, we concluded that they did not influence the non-canonical pathway. The expression level of phosphorylation of NF-κB was supported with qPCR, which showed the LPS-enhanced NF-κB expression, which was suppressed by pre-treatment of BGG (Figure 8C). These findings indicated that pre-treatment with BGG downregulates the LPS-induced NF-κB activation, p65, and p50 nuclear translocation, leading to inflammation.

**Figure 12:**
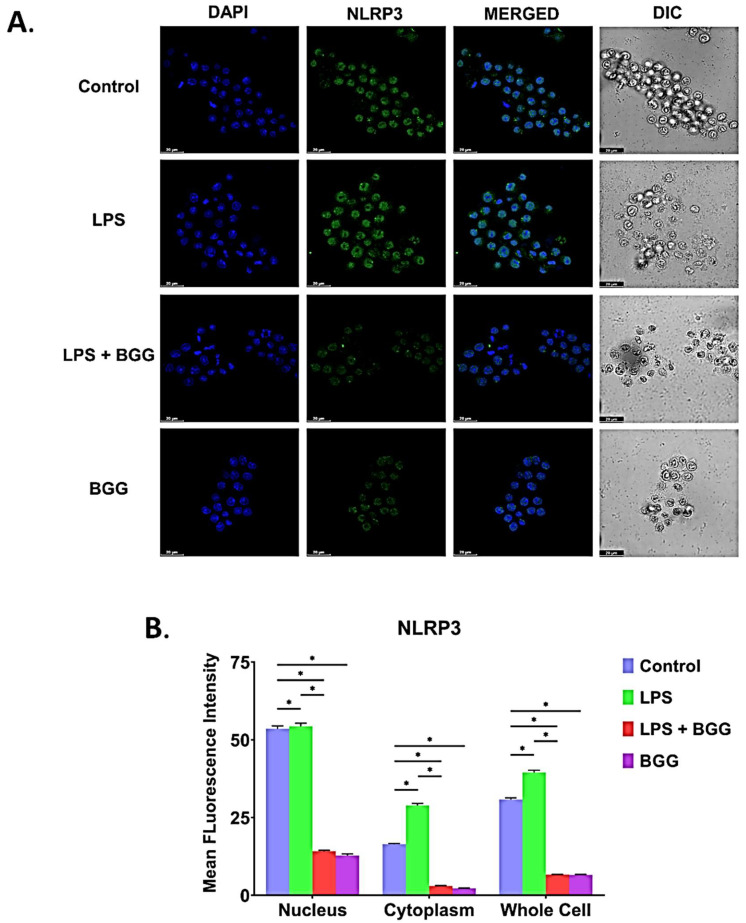
Immunofluorescence staining images representing the BGG attenuation of the LPS-enhanced translocation of NLRP3 from the cytosol to the nucleus measured by confocal microscopy. (**A**) The translocation assay of NLRP3 in RAW 264.7 macrophages. (**B**) The mean fluorescence intensity showing the NLRP3 ratio of nucleus vs. cytoplasm. Results are mentioned as mean ± SEM (*n* = 3 independent experiments) (* *p* ≤ 0.05). Scale bars: 10 µm.

**Figure 13:**
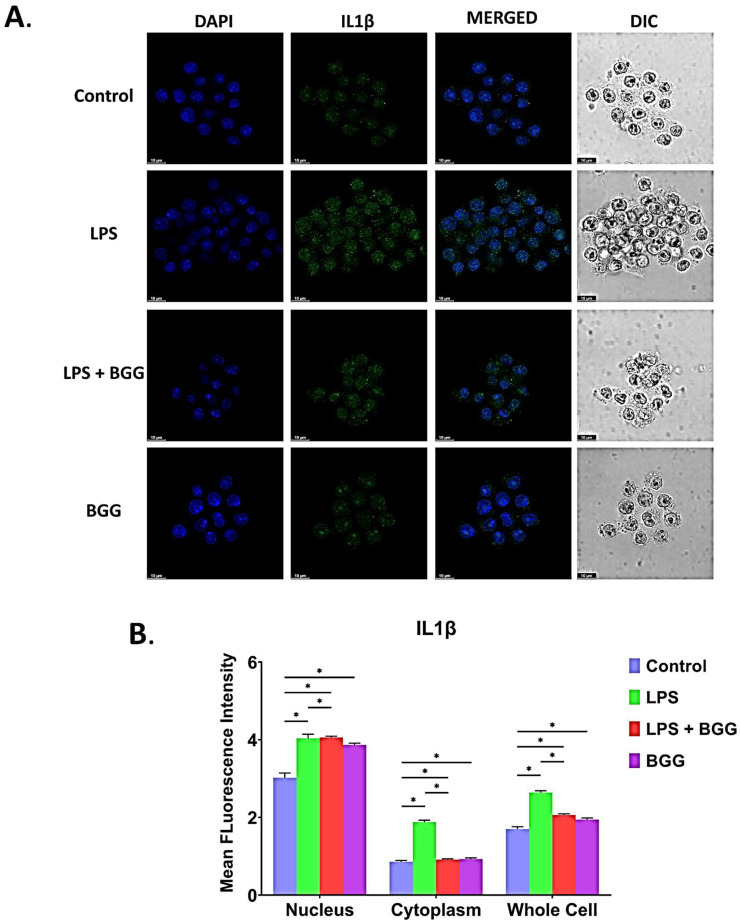
Immunofluorescence staining images representing the BGG attenuation of LPS-induced inflammasomes in the RAW 264.7 cells by analyzing the expression of IL-1β measured by confocal microscopy. (**A**) The accumulation and condensation of IL-1β in RAW 264.7 macrophages. (**B**) The mean fluorescence intensity showing the IL-1β in the cytoplasm, nucleus, and whole cells. Results are mentioned as mean ± SEM (*n* = 3 independent experiments) (* *p* ≤ 0.05). Scale bars: 10 µm.

We have shed light on the formation of NLRP3 inflammasomes, the main pathway involved in pyroptosis. The formation of the NLRP3 inflammasome occurred in two ways; the NF-κB signal pathway, which is started with LPS treatment, and the inflammasome activation pathway. The massive intracellular NLRP3 inflammasome accumulation in the cytosol indicates pyroptosis (inflammation-induced apoptosis). We showed suppressive effects of BGG on NF-κB signaling; hence we further analyzed whether NLRP3 inflammasome is involved in the anti-inflammatory effects of BGG. To investigate this process, the cells were pre-treated with BGG (10 μΜ) for 1 h and were treated with LPS (1 μg/mL) for 23 h and ATP (5 mmol) for 1 h. The level of NLRP3 inflammasome and IL-1β were evaluated by the immunofluorescence staining, which revealed that expression of these markers was significantly higher in LPS and ATP treatment, which was conspicuously suppressed by pre-treatment with BGG (Figure 12A,B). Immunofluorescence staining showed that, upon LPS and ATP stimulation, the NLRP3 inflammasome and IL-1β were more condensed in the RAW 264.7 cells. Pre-treatment with BGG significantly suppressed the LPS and ATP-induced NLRP3 inflammasome complex formation, IL-1 beta expression, and pyroptosis process (Figure 13A,B).

### 2.9. Molecular Docking

Molecular docking of BGG was performed against TLR4. BGG showed binding energy of −7.0 kcal/mol with TLR4 protein. The binding and modes of interaction of BGG with this protein were shown in Figure S5A, and the interacting amino acids were listed in Figure S5B. In TLR4, the BGG showed conventional hydrogen binding with PHE:481, ASN:484, GLU:507, ASN:529, ASN:528, and LYS:482, at the same site of the LPS binding pocket.

### 2.10. BGG Relieves LPS-Induced Inflammation and Toxicity In Vivo Sepsis Model

**Figure 14:**
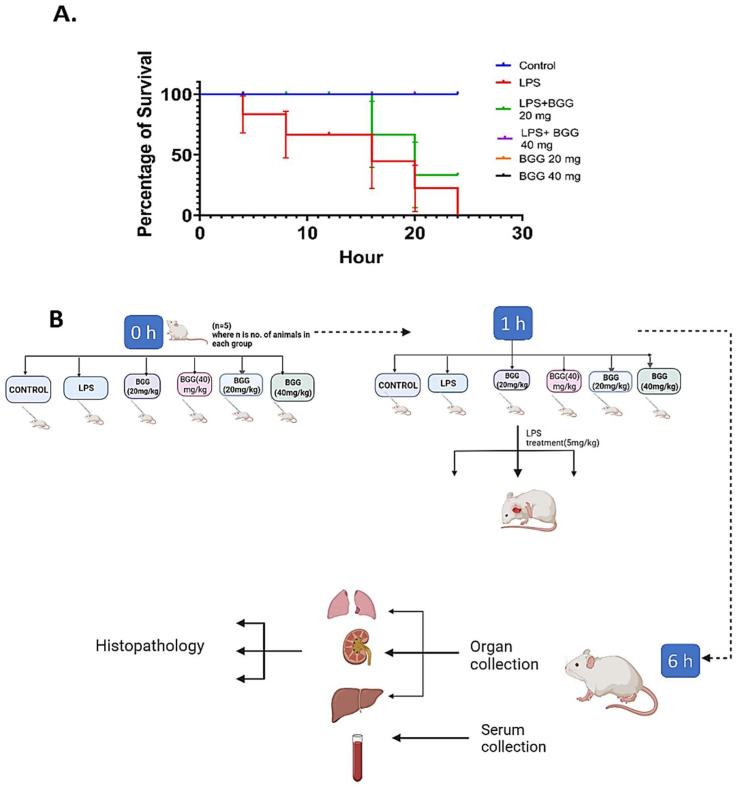
Representation of the BGG protection on LPS-induced sepsis model in mice. (**A**) Animal survival data. (**B**) Flow charts depict the LPS-induced sepsis model in mice.

BGG showed the ability to combat LPS-induced inflammation at the cellular level. Sepsis is mainly characterized by the induction of various inflammatory cytokines and dysregulated systemic host response [24,25]. Further analyzing the protective activity of BGG in vivo, we prepared the murine model with systemic inflammation similar to sepsis. The mice (*n* = 6) were pre-treated intraperitoneally to examine the animal survival rate with two doses of the BGG, i.e., 20 mg/kg and 40 mg/kg for 1 h, followed by LPS (5 mg/kg) for 23 h. All the mice died in the LPS dose group, two mice died in LPS + BGG 20 mg/kg, and none died in the LPS + BGG 40 mg/kg (Figure 14A). Furthermore, to undertake this consideration, we injected the two different doses of BGG (20 mg/kg) and (40 mg/kg) into BALB/c mice intraperitoneally 1 h before intraperitoneal injection of LPS (5 mg/kg) for 6 h. After 6 h of LPS challenge, the mice were sacrificed to collect the liver, kidney, lung tissues, and blood serum (Figure 14B). Mice pre-treated with BGG showed less inflammatory cell infiltration and tissue degradation as observed in H&E staining than in the LPS-treated mice group.

#### 2.10.1. Liver

**Figure 15:**
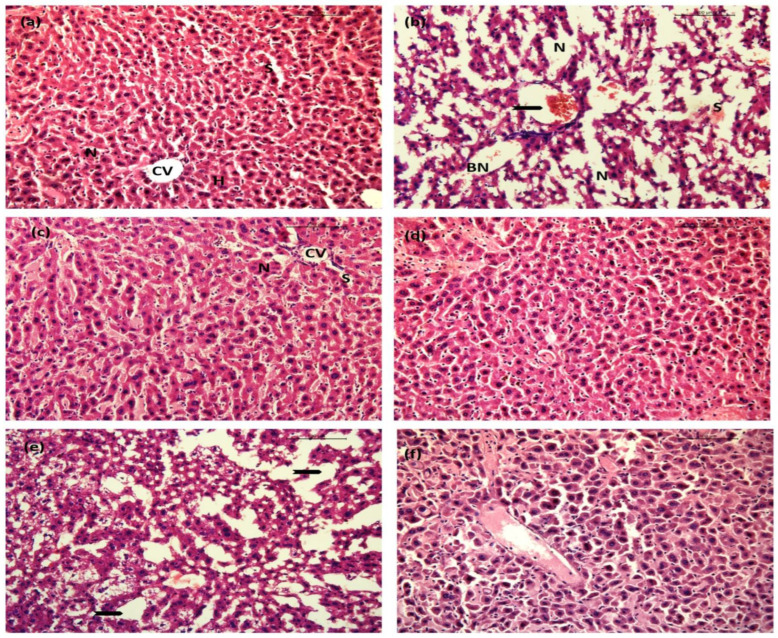
Images represent the BGG protection on LPS-induced sepsis model in mice. Hematoxylin and eosin (H&E)-stained liver. (**a**) Saline showing normal hepatic architecture with the central vein (CV) and surrounding hepatocytes (H), sinusoids (S), and nucleus (N). (**b**) Lipopolysaccharide (LPS) endotoxin showing hepatic mononuclear cell infiltration, congestion of central vein and hemorrhage (arrowhead) and blood sinusoids (S), binucleated hepatocytes (BN), necrotic area (N), congested blood sinusoids (S). (**c**,**d**) BGG 20 mg/kg and 40 mg/kg showing no significant pathological alteration. (**e**) LPS + BGG 20 showing moderate degeneration of hepatic architecture with necrotic areas (arrowhead). (**f**) LPS + BGG 40 indicating least necrotic and scarred areas with mild blood congestion.

The liver section of saline-treated mice exhibited normal hepatocytes with intact cellular morphologies with a prominent central vein, distinct nucleus, and hepatocyte density (Figure 15a). LPS intoxication led to various histological deformities, including congestion of the hepatic portal vein, centrilobular necrosis, inflammatory cell infiltration, and massive hemorrhagic necrosis. Also, histopathological examinations revealed sinusoid congestion with necrotic areas (Figure 15b). No significant (visual) changes were observed in the liver sections of *perse* groups receiving BGG treatment at two different doses (BGG 20 mg/kg and 40 mg/kg) compared to the control group. No change was observed in hepatocyte density, structure, or size. The hepatic portal vein looked similar to the control group. No vacuoles or increased extracellular spaces were observed (Figure 15c,d). Attenuating histological abnormalities were observed in the LPS + BGG 20 pre-treatment group by reducing tissue necrosis (Figure 15e). Other LPS + BGG 40 treated group (Figure 15f) showed minor hepatological degeneration with mild blood congestion and the least scarred tissue.

#### 2.10.2. Kidney

**Figure 16:**
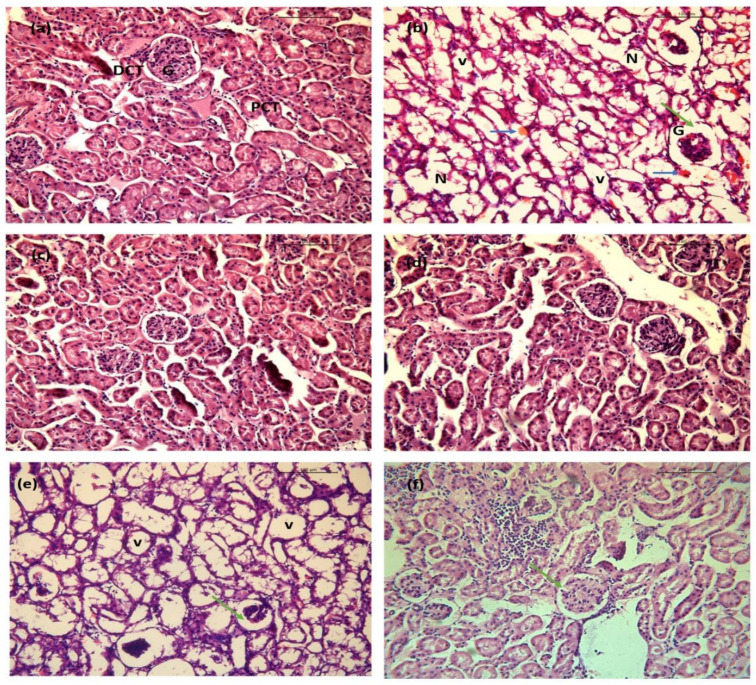
Images represent the BGG protection on LPS-induced sepsis model in mice. Hematoxylin and eosin (H&E)-stained kidneys. (**a**) Saline showing normal morphological structure with glomerulus (G), proximal convoluted tubules (PCT), and distal convoluted tubules (DCT). (**b**) Lipopolysaccharide (LPS) endotoxin showing severe degenerative alterations with tubular necrosis (N), vacuolation of renal tubules (V), enhancement of Bowman’s space (green arrow), hemorrhage in interstitial tissue (blue arrow). (**c**,**d**) BGG 20 mg/kg and 40 mg/kg showing no significant pathological alteration. (**e**) LPS + BGG 20 showing moderate tubular degeneration with reduction of Bowman’s space (green arrow). (**f**) LPS + BGG 40 showing the normal glomerular structure and Bowman’s space with mild vacuolation of renal tubules.

The protective effects of BGG on LPS-induced renal injury were investigated by histological examination of kidney tissues. Figure 16a illustrates the normal morphological parameters of the control group with normal glomerular and tubular structures with no evidence of injury. In contrast to the control group, the LPS-treated group showed significant cellular injuries (Figure 16b). The main alterations included Bowman’s space increment, multiple tubular vacuolization areas, glomerular ischemia, hemorrhage in interstitial tissue with hemosiderosis, and loss of brush border and tubular dilation. Histological analysis of *perse* groups demonstrated no inflammatory or cellular alterations (Figure 16c,d), indicating that BGG alone is not stressful to different organs. Debilitation of renal injury with BGG pre-treatment was observed at a dose of 20 mg/kg with a lessening of Bowman’s space and a reduction in the glomerular capillary wall (Figure 16e). Further improvement in renal tissue injury was observed in the LPS + BGG 40 group with mild vacuolation of renal tubules and close to the normal histological architecture of the kidney (Figure 16f).

#### 2.10.3. Lung

**Figure 17:**
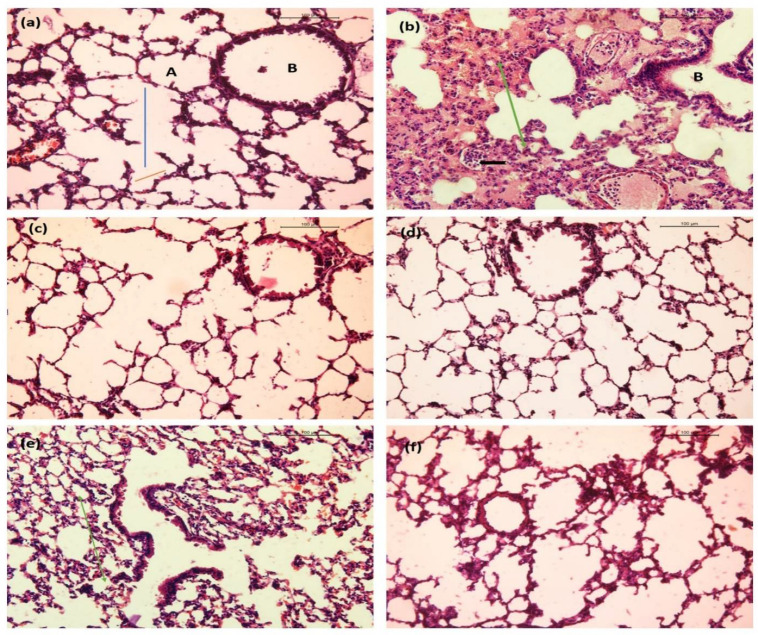
Images represent the BGG protection on LPS-induced sepsis model in mice. Hematoxylin and eosin (H&E)-stained lung sections from mice treated with (**a**) saline showing normal and distinct respiratory bronchiole (B), alveolus (A), alveolar duct (blue line), and alveolar sac (red line). (**b**) Lipopolysaccharide (LPS) endotoxin showing severe degenerative alterations in the lung with ruptured bronchiole (B), interstitial and intraalveolar hemorrhage and alveolus atelectasis and fusion (green arrow), inflammatory cell infiltration (arrowhead). (**c**,**d**) BGG 20 mg/kg and 40 mg/kg showing no significant pathological alteration. (**e**) LPS+ BGG 20 showing moderate alveolar damage with reduction of interalveolar septum thickening (green arrow). (**f**) LPS + BGG 40 showing mild thickening of some alveolar-capillary with intact reparatory bronchiole. Scale bars: 100 µm (**f**) Measuring extracellular cytokine interleukins by CBA bead assay analyzed by using flow cytometry in mice serum.

A histological analysis of lung tissue from the control group demonstrated normal structure with distinct respiratory bronchiole and no histopathological changes under a light microscope (Figure 17a). Lung histological sections of LPS-challenged mice exhibited thickened alveolar walls with alveolus atelectasis and fusion, less alveolar space and interstitial congestion, and an accumulation of a large number of neutrophils in the intra- and interalveolar space (Figure 17b). Lung sections of BGG groups were similar to that of a control group. Terminal bronchioles were distinctively visible with no deformities. Alveolar ducts and alveoli were normal (Figure 17c,d). Reduction in tissue injury in the LPS + BGG 20 pre-treatment group was observed compared to the LPS group, with a decrease in interalveolar septum thickening (Figure 17e). LPS-induced pathological changes were significantly attenuated in LPS + BGG 40 group with a substantial reduction in pulmonary edema with intact reparatory bronchiole (Figure 17f).

#### 2.10.4. BGG Reduces the LPS-Induced Inflammatory Response in the Sepsis Model

**Figure 18:**
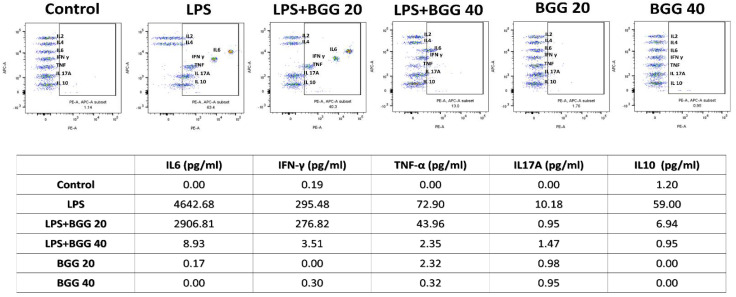
Measuring extracellular cytokine interleukins by CBA bead assay analyzed by using flow cytometry in mice serum.

In vivo anti-inflammatory activity of BGG was evaluated in the BALB/c sepsis model. As shown in Figure 18, the in vivo study showed a significant increase in IL-6, IFN-γ, TNFs, IL-17A, and IL-10 expression in serum of LPS (5 mg/kg)-treated mice, and no considerable changes in IL-2 and IL-4 levels were observed. The treatment with 20 mg/kg of BGG showed a mild decrease in IFN-γ and TNFs levels, whereas when treated with 40 mg/kg of BGG, the IFN-γ and TNFs levels showed a significant reduction in serum of previously LPS-treated mice. BGG treatment in both 20 mg/kg and 40 mg/kg concentrations showed a significant decrease in IL-6, IL-17A, and IL-10 levels in serum, but a 40-mg/kg concentration of BGG showed a higher neutralization ability than a 20-mg/kg concentration of BGG. No significant change was observed in serum levels of IL-6, IFN-γ, TNFs, IL-17A, and IL-10 in mice treated with BGG at 20 mg/kg and 40 mg/kg without LPS stimulation.

### 2.11. GSH Assay

BGG treatment increased the total GSH concentration in cell lysate samples, and we have observed a dose-dependent, significant increase in GSH concentration in the mice serum sample. On the other hand, LPS lowered GSH concentration in mice serum and cell lysate samples (14.88 µM and 10.13 µM). We have also observed a significant increase in the GSH concentration in the pre-treated BGG samples, which were later subjected to LPS, concerning only LPS-treated samples (13.60 µM in cell lysate, 15.83 µM, and 18.7 µM in animal serum. Interestingly, BGG treatment alone also enhanced the GSH activity in the control experiment (13.43 µM to 15.45 µM in cell lysate and 19.722 µM to 21.49 µM and 23.78 µM) (Figure S5C,D).

## 3. Discussion

Due to fewer long-term side effects, many natural products like terpenoids, alkaloids, flavonoids, and glycosides play a significant role in human health, especially in managing chronic inflammation. β–glucogallin (1-O-galloyl-β-d-glucopyranose), a plant-derived polyphenolic ester, is believed to protect due to its free radical scavenging property against several diseases like diabetes, glaucoma, hepatic damage, skin damage from UV, etc. [4,5,6,26]. It also upregulates anti-oxidant enzymes GSH, CAT, and SOD and acts as an aldose reductase inhibitor. Thus, it could be a novel therapy against diabetic complications such as cataracts [8]. The macrophage, the primary cell of the innate immune system, is considered to play a protagonist role in modulating inflammatory responses. They are also found in tissues as resident cells survey their surroundings and remove invading pathogens, apoptotic cells, and debris, thus maintaining tissue integrity [27]. BGG, like other natural compounds that act as anti-oxidants, barely show any activity on normal macrophages (Figure S1B). LPS is a potent activator of macrophages. LPS signaling leads to the early activation of NF-κB, IRF3, and MAPK kinase pathways, leading to numerous pro-inflammatory genes [13]. In this study, we investigated the effects of BGG on the LPS-stimulated murine macrophage cell line, RAW 264.7, on the production of pro-inflammatory mediators (NO and COX2) and cytokines (IL-6 and TNF, IFN) and also on an LPS-induced in vivo sepsis model as described by Haimei Tang et al. [25]. Performing gene expression profiling, we have also tried to detail the mechanism of the anti-oxidant effects of BGG.

Activated macrophages undergo respiratory bursts and are used for examining the anti-oxidant potential of natural anti-oxidants in pharmacology [28]. Reactive oxygen species are produced in cells through many sources. It is produced intentionally as part of a signaling pathway, defense mechanism, or molecular synthesis process and as by-products of various biological processes. ROS are produced and released by macrophages in response to phagocytosis [29]. Apart from phagocytes, non-phagocytic cells have recently been shown to produce ROS enzymatically in response to inflammation and tissue damage. NADPH oxidase is the first source of ROS that has been identified in macrophages [30]. Mitochondria are responsible for primary ROS production in the form of superoxide, which is generated when molecular oxygen is reduced by leaking electrons, as electrons flow down the electron transport chain, resulting in superoxide anion accumulation in the mitochondria [31]. BGG enters the cells by the natural diffusion process and is responsible for scavenging the reactive oxygen species, reactive nitrogen species, etc., inside the activated macrophages. BGG in the LPS-induced macrophages also restore mitochondrial membrane potential, as evident by JC-I staining and MitoSox staining. The effect of BGG on the protection of ROS production was almost equivalent to that of known anti-oxidant N-acetyl cysteine (NAC) (Figure 1A–C). The anti-oxidant property of NAC is primarily due to its ability to reduce glutathione to GSH, a well-known anti-oxidant and a substrate of several anti-oxidant enzymes [32]. Our data showed that pre-treatment with BGG increased cellular reduced glutathione (GSH) levels (Figure S5C,D).

LPS and other inflammatory cytokines, including IL-1β, TNF-α, and IFN-γ, produce larger amounts of NO for longer [33]. NF-κB is also regarded as an inducer of iNOS at the transcription level. Upon reaction with a superoxide (O_2−_), NO becomes cytotoxic peroxynitrite (ONOO^−^), which consequently exerts microbicidal effects. By cytotoxicity and oxidative stress, NO overproduction results in cell death and tissue death. In addition, iNOS is a harmful enzyme involved in the pathogenesis of inflammation. Several NOS inhibitors were developed, but most of them could not move past the clinical trials due to their toxicity [34]. Thus, the search for non-toxic and potent iNOS inhibitors from natural products has come up with a very high priority. Many natural products like quercetin, (−)-epigallocatechin gallate, (+)-catechin, etc., showed iNOS inhibitory action either via blocking NF-κB activation or by inhibition of NF-κB-IL6 activation [35]. Moreover, COX-2 is a key factor for synthesizing PGs and mediates inflammatory responses resulting in fever, pain, and hypersensitivity [36]. Our study also found similar iNOS inhibitory action of BGG on LPS-induced inflammation in RAW 264.7 macrophage cells. Therefore, we believe that BGG could be a potent iNOS inhibitor that could be used for a clinical trial.

Gene expression analysis achieved an evaluation of the effect of BGG on LPS-activated RAW 264.7 macrophage cells at a transcriptional level. The pathway deduced from the upregulated and downregulated gene expression mainly involved inflammatory, cell death, and metabolic pathways. From further study, we inferred that BGG acts through the TLR4 transduction pathway. LPS activates TLR4, and TRIF-dependent acute inflammation occurs at a late stage, while the MyD88-dependent pathway occurs at an early stage of acute inflammation [37]. The recruitment of IL-1R-associated kinases 1 (IRAK1), IRAK2, IRAK4, and TNF receptor-associated factor 6 (TRAF6) is required to activate the MyD88-dependent pathway and activate the mitogen-activated protein kinase (MAPK) [38]. Activation of MAPKs is followed by activating a transcription factor c-fos belonging to the activator protein-1; c-fos binds to c-jun and are translocated in the nucleus. There c-jun/c-fos produces Fas and Fas ligand, a death domain-containing member of the TNF receptor family and matrix metalloproteinase, especially MMP-9. Fas/Fas-L, which leads to apoptosis [39]. Apart from this, a MyD88-dependent pathway is used to bridge the MAL(TRIF) pathway, leading to phosphorylation of TRAF6, which activates the mitogen-activated protein kinase (MAPK) kinase (IκB) complex. The activation of IKKα leads to the phosphorylation of IκBα, which can then be polyubiquitinated and degraded, leading to the transfer of NF-κB from the cytosol to the nucleus [40]. NF-κB is responsible for iNOS production and cytokine production, such as IL-6, TNF-α, IL-17, IL-1β, IL-4, and IL-10. Upon reaction with a superoxide (O_2_^−^), NO becomes cytotoxic peroxynitrite (ONOO^−^), which consequently exerts microbicidal effects. By cytotoxicity and oxidative stress, NO overproduction results in cell death and tissue death [41]. In addition, iNOS is a harmful enzyme involved in the pathogenesis of inflammation [36]. Our present study showed that BGG inhibited the TLR4 pathway and decreased NF-κB activity in LPS-activated RAW 264.7 cells (Figure 8). As a result, the production of pro-inflammatory cytokines (TNF-α, IL-6, IL-1β) and mediators (NO, COX2) were suppressed. Molecular docking analysis also revealed that the BGG binds at the LPS binding pocket at TLR4 protein (Figure S5A). Thus, BGG attenuates the LPS-driven activation signaling in macrophages.

The NF-κB, an important transcription factor, influences the expression of pro-inflammatory genes, including cytokines, chemokines, and adhesion molecules. NF-κB has long been considered an essential anti-inflammatory drug target. The NF-κB signaling pathway plays an important role in the inflammatory process. It regulates gene expression related to immune responses and cell survival, such as TNF-α, interleukin IL-1β, and IL-10 [16,17]. In an unstimulated state, NF-κB is presented in the cytosol, combined with inhibitory protein IκB. Specific stimuli such as LPS give rise to free NF-κB via the degradation of IκB through phosphorylation by IκB kinase (IKK). Activated NF-κB translocates from the cytoplasm to the nucleus, binds to the promoter, and modulates the expression of inflammatory genes, including iNOS, inflammatory cytokines, and chemokines [42]. Most anti-inflammatory drugs have been shown to repress the expression of inflammatory mediator genes by inhibiting the NF-κB activation pathway. Our data confirm that BGG blocked the translocation of activated NF-κB to the nucleus, and thus it blocks an LPS-induced stimulatory signature (Figure 10 and Figure 11). Therefore, it may be a suitable candidate to be an NF-κB inhibitor.

Inflammation is a complex reaction to body aggression by a pathogen agent, an allergen, a toxic compound, a tissue lesion, etc., where innate immune cells like macrophages play vital protective roles by triggering the immune responses against infection. Inflammation acts as a protective mechanism, but uncontrolled inflammation reactions may develop chronic diseases like sepsis, IBD, etc. LPS activates macrophages to initiate an inflammatory response to secrete pro-inflammatory cytokines such as tumor necrosis factor-α (TNFα), interferon-gamma (INFγ), interleukin 6 (IL-6), response to inflammation [43]. By suppressing the productions of IL-6, TNF, and IFN-gamma, BGG showed primarily anti-inflammatory effects on an LPS-stimulated RAW 264.7 macrophage cell line and in an vivo mice model. Several classes of natural products like flavonoids (quercetin, rutin, catechin), polyphenols, and diterpenes (andrographolides) showed anti-inflammatory actions by iNOS inhibition either via blocking NF-κB activation or by inhibition of IL6 activation [44].

Inflammasomes are multi-protein oligomers that are critical for host immune defenses. Inflammasomes are primed by NLRP3 production due to bacterial LPS binding to TLR4, leading to the nuclear localization of activated NF-κB, which promotes the transcription of NF-κB-dependent genes, such as NLRP3, and Pro-IL-1β, which are eventually necessary for inflammasome activation [45]. Inactivated macrophages, such as the NLRP3 inflammasome, have been regarded as regulators in the pathogenesis of multi-inflammatory diseases like atherosclerosis, sepsis, etc. [46]. For this reason, we have checked the role of BGG on LPS-induced inflammasomes in macrophages. We also confirmed that BGG reduced NLRP3 productions and NLRP3 inflammasome activation-induced IL-1β production (Figure 12 and Figure 13).

LPS can cause sepsis in mice with the devastating spurt of pro-inflammatory cytokines, increasing the number of inflammatory mediators and inflammatory cells [34,35]. Uncontrolled inflammation may result in dysregulation of the immune system and eventually life-threatening tissue damage and multiple organ failure. Sepsis has a concerning yearly mortality rate (15–19 million worldwide), making the disease one of the major causes of morbidity [24]. Natural products like berberine, curcumin, and many herbal drugs have significant potential against sepsis [47]. Pre-treatment with BGG had a protective effect in controlling pro-inflammatory cytokines, and tissue damage in the anti-LPS-derived sepsis model. Pre-treatment with 40 mg/kg BGG gave 100% protection against the LPS-derived sepsis model. To our knowledge, this has been the first report of BGG’s role against sepsis.

**Figure 19:**
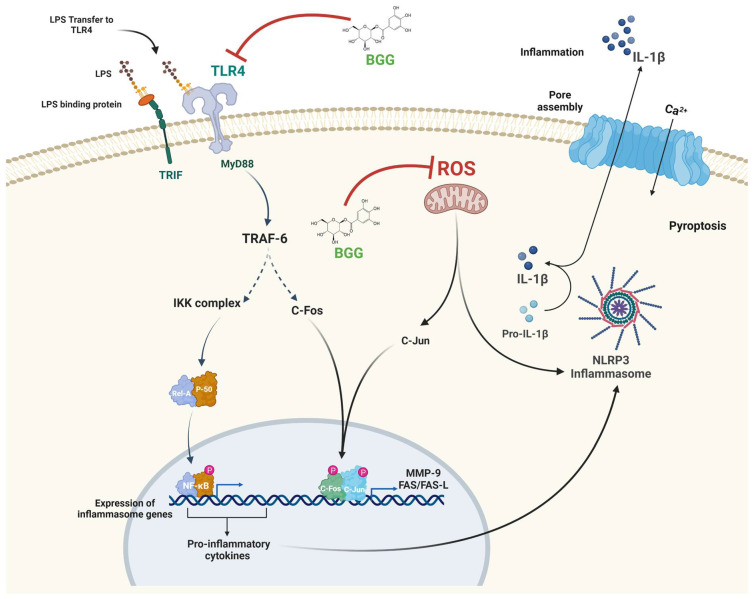
Represents the predicted protective pathway of BGG for blocking LPS signalling.

We conclude that BGG suppressed LPS-induced ROS, NO, and pro-inflammatory cytokines and their mediators via inhibiting TLR4, NF-κB signaling pathways, and NLRP3 inflammasomes in a macrophage cell line (Figure 19). BGG also protects against LPS-induced sepsis-related cell damage and blocks inflammatory cytokine production in these mice. Thus, BGG could be a potent anti-inflammatory compound and iNOS inhibitor again.

## 4. Methods and Results

### 4.1. Chemicals and Reagents

β-glucogallin (BGG, molecular formula-C_13_H_16_O_10_) (Cat. G416000) (Figure 1A) was obtained from the Toronto Research Chemicals (Toronto, ON, Canada). All cell culture reagents are obtained from the Gibco-BRL (Life science technologies). DCFDA (D6883), LPS (from *E. coli* O111:B4) (L4391-1MG), protease inhibitor cocktail (P8340), and Griess reagent (G4410-10G) were purchased from Sigma Aldrich, St. Louis, MI, USA. BCA protein assay reagent (23225), DAPI (D1306), and Mitosox red (M36008) were purchased from Thermo scientific. ECL was obtained from biorad, JC-1 (T3168). Rabbit antibodies for p-p65 (Ser536, 1:200) (3033S), p-p50 (12540S, 1:200), RelB (3017T, 1:200), and Alexa Fluor 488 Anti-rabbit (4412S, 1:500) were purchased from cell signaling. NLRP3 (IMG-6668A, 1:200) and IL-1 beta (NB600-633, 1:200) were purchased from Novus biologicals.

### 4.2. Cell Culture and Cytotoxicity Determination

RAW 264.7 cells were grown in high-glucose DMEM containing 10% fetal bovine serum at 37 °C with 5% CO_2_. BGG cytotoxicity for RAW 264.7 cells and mice peritoneal was evaluated by MTT assay. Briefly, 9 × 10^3^ RAW 264.7 cells were plated into 96-well plates and kept at 37 °C overnight with 5% CO_2_. The cells were treated with different concentrations of BGG (1, 5, 10, 20, 40, 60, 80, 100 μM), followed by incubation for 24 and 48 h. To analyse the protective effect of BGG, RAW 264.7 and mice peritoneal cells were plated into 96-well plates. BGG (10 μM) was pre-treated in RAW 264.7 and mice peritoneal cells, followed by the LPS (1 μg/mL) for 24 and 48 h. To analyse the cell viability, 10 μL of 5 mg/mL of MTT was added to RAW 264.7 and mice peritoneal macrophage cells for 2–3 h at 37 °C.

### 4.3. Determination of NO Production

Nitric oxide production in the culture medium of RAW 264.7 cells was determined by using Griess reagent (2% sulphanilamide, 0.2% N-(1-naphthyl) ethylenediamine dihydrochloride in 5% phosphoric acid). For prediction of BGG-induced nitrite production, 1 × 10^5^ RAW 264.7 cells were seeded in 12-well plates for 24 h, followed by treatment with various concentrations of (1, 5, 10, 20, 40, 60, 80, 100 μM) BGG without LPS stimulation. Furthermore, to analyze the effect of BGG on LPS-stimulated Raw 264.7 cells and mice peritoneal macrophages, the 1 × 10^5^ cells were plated in 12-well plates. The 10 μM of BGG was treated for 1 h before *Escherichia coli* lipopolysaccharide (LPS) (1 µg/mL) stimulation for 24 h. The media (100 μL) was mixed with an equal amount of Griess reagent in 96-well plates. After 10 min incubation at room temperature in the dark, the absorbance was measured at 550 nm by using the Synergy H1 hybrid multimode microplate reader.

### 4.4. Reactive Oxygen Species Detection

To study the total ROS generation of BGG, 1 × 10^5^ RAW 264.7 cells were seeded into 12-well plates and incubated for 24 h, followed by adding various concentrations of BGG without LPS stimulation (1, 5, 10, 20, 40, 60, 80, 100 μM). Furthermore, to analyze the effect of BGG on LPS-induced total ROS production, the 1 × 10^5^ RAW 264.7 and mice peritoneal macrophages cells were seeded into 12-well plates. The pre-treatment was performed with 10 μM of BGG to the plates for 1 h after treatment with LPS (1 μg/mL). After 24 h incubation, a sensitive fluorescent probe DCFH-DA was added in a final concentration of 10 μM to each well and incubated for 30 min at 37 °C. After staining, the cells were washed and analyzed by flow cytometry.

### 4.5. miRNA Microarray Analysis

A miRNA microarray analysis was performed to identify the miRNA expression profiles of the extracted RNA samples. Total RNA was isolated by using TRIzol reagent (Molecular Research Center, Inc., Cincinnati, OH, USA) and was purified to obtain the miRNA fraction, further purified by using a mirVana miRNA isolation kit (Ambion, Austin, TX, USA) according to the manufacturer’s instructions. The isolated samples were labeled with Hy3 by using the miRCURY array labeling kit (Exiqon, Vedbaek, Denmark) and hybridized with miRCURY-locked nucleic acid (LNA) microRNA arrays (v8.0; Exiqon). After hybridization, microarray images were taken with a Genepix 4000B scanner (Axon Instruments, Foster City, CA, USA) and analyzed with Genepix Pro 6.0 software (Axon Instruments). Data normalization was performed by using the quantiles method. We have selected the TLR4, FOS, FAS, MMP9, and MAPK pathways based on miRNA analysis. The qPCR confirmed these pathways.

### 4.6. Evaluation of Mitochondrial Membrane Potential (ΔΨ)

JC-1 fluorochrome was used to evaluate the depolarization in mitochondrial membrane potential. Untreated and treated cells were washed and stained with 10 μM JC-1 in the dark at 37 °C for 20 min, following the manufacturer’s instructions. After staining, cells were analyzed by flow cytometry and confocal imaging. When mitochondria are intact in a cell, it produces red fluorescence indicated by FL2-H; the cells with depolarized mitochondria have green fluorescence shown by FL1-H by using software (Becton Dickinson, Franklin Lakes, NJ, USA). The LPS induced the significantly enhanced J-monomers and decreased the J-aggregates.

### 4.7. Evaluation of Mitochondria Produced Superoxide

To analyze the protective effect of BGG on LPS-induced superoxide production in RAW 264.7 cells, the 1 × 10^5^ cells/well were seeded into 12-well plates, and pre-treatment was performed with BGG (10 µM) and NAC (10 µM) for 1 h, followed by LPS (1 μg/mL) stimulation for 23 h. After treatment, cells were harvested and stained with MitoSOX^TM^Red with a concentration of 5 μM for 30 min at 37 °C. After staining, the cells were washed and analyzed by flow cytometry.

### 4.8. Cytometric Bead Array (CBA) for Extracellular Cytokines

A BD CBA mouse Th1/Th2/Th17 cytokine kit was used to check the levels of Th1 (IL-2, TNF, and IFN-α), Th2 (IL-4, IL-6, and IL-10), and Th17 (IL-17 A) cytokines according to the manufacturer’s instructions. A total of 50 µL of RAW macrophages, mice peritoneal macrophages suprenant, and animal serum sample were mixed with 50 µL of capture beads, loaded into the assay tubes, and incubated in the dark for 150 min. After adding 300 µL of wash buffer, samples were analyzed via BD LSR Fortessa flow cytometry.

### 4.9. Intracellular IL6, IFN, and IL10 Production

According to previous studies, flow cytometry determined intracellular IL-6, IFN, and IL-10 levels following manufacturer instructions [48]. The data were collected on an LSR-Fortessa flow cytometer (BD, Franklin Lakes, NJ, USA) and analyzed with FlowJo software.

### 4.10. Quantitative Real-Time PCR (qPCR) Analysis

The total mRNA from the RAW 264.7 macrophages was extracted by Trizol reagent. The RNA extraction from the cell samples was further transcribed into cDNA by using a Biorad one-script cDNA synthesis kit. SYBER-Green was used to evaluate the expression of all genes mentioned in Table 1.

### 4.11. Animals and Treatments

Weight-normalized mice were randomized into 6 groups (*n* = 6): (1) control group, (2) LPS group (5 mg/kg), (3) BGG + LPS group (20 mg/kg; 5 mg/kg), (4) BGG + LPS group (40 mg/kg; 5 mg/kg), (5) BGG group (20 mg/kg), (6) BGG group (40 mg/kg). BGG was administered to mice by the intra-peritoneal route. In the present study, after 1 h of BGG administration, a single systemic dose of LPS (5 mg/kg, i.p.) was administered to mice as systemic administration of LPS triggers acute inflammation. Afterward, mice were anesthetized by isoflurane and decapitated after 6 h of LPS treatment. Major organs like the liver, lungs, spleen, and kidneys were collected immediately and were stored in 10% phosphate-buffered formalin for histological analysis.

### 4.12. GSH Assay

According to the manufacturer’s instructions, the reduced and total GSH in mice serum and cell lysate was determined by using the fluorescent detection kit K006-F5 (Arbor Assays, Ann Arbor, CA, USA).

### 4.13. Isolation of Peritoneal Macrophages

The liver, spleen, gastrointestinal system, and other viscera are housed in the peritoneal cavity, a membrane-bound and fluid-filled abdominal cavity seen in mammals. Because the peritoneal cavity has many naive macrophages, it is an excellent place to collect naive tissue-resident macrophages. The euthanized mouse was sprayed with 70% ethanol and mounted on a Styrofoam block; 5 mL of ice-cold PBS was injected into the peritoneal cavity, followed by a gentle massage to dislodge the attached cells into the PBS solution. The fluid was collected with a needle attached to a 5-mL syringe from the peritoneum. The cell suspension was spun at 500× *g* for 8 min and washed with PBS. The cell obtained was primarily cultured with DMEM high glucose [49].

### 4.14. Molecular Docking of BGG against TLR4

Molecular docking is a widely used tool for analyzing ligands’ binding affinity and interaction with protein molecules [50,51]. Molecular docking of the BGG was performed by utilizing the PyRx version 0.8 with Autodock vina with a Lamarckian genetic algorithm and an empirical free energy force field [52]. The crystal structure of TLR4, with the SDF file of BGG, was loaded to the PyRx virtual screening software. The protein was considered a rigid molecule during the process, and BGG was considered a flexible molecule. The ligand-protein interaction was analyzed by by using BIOVIA Discovery studio visualizer version, 2016.

### 4.15. Histological Analysis

Histopathology is the study of analyzing the signs of the disease by using the microscopic techniques of a biopsy or surgical specimen fixed onto glass slides. To visualize different components of the tissue and organs under a microscope, the sections of the tissues are coated or dyed with one or more stains. Four percent paraformaldehyde was used to fix tissue samples by using a neutral buffer solution for 24 h, followed by gradual dehydration with different ethanol concentrations. Afterward, they were taken to a tissue-embedding station, where tissues were placed into embedding molds with the aid of molten paraffin. Serial sections of 5 µm thickness were cut by using a microtome and fixed in glass slides coated with egg albumin: Glycerol (1:1) with a mix of thymol crystal, followed by hematoxylin and eosin (H&E) staining [53].

### 4.16. Immunofluorescence Staining

To analyze the expression of p-65 (1:200 dilution), p-50 (1:200 dilution), RelB (1:200 dilution), translocation, and inflammasome formation detection by NLRP3/IL-1β (1:200 dilution). The RAW 264.7 macrophage cells were seeded overnight on a 35-mm high glass-bottom dish. As described above, cells were pre-treated with BGG, followed by LPS (1 μg/mL). Cells were fixed with 4% paraformaldehyde and permeabilized by using 0.2% Triton X-100 in PBS. Cells were incubated with primary antibodies overnight, washed with PBS to remove the excessive primary antibodies, followed by incubation with fluorescent secondary antibodies, and Alexa Fluor 488 Anti-rabbit (4412S, dilution 1:500) [54]. The cell’s nucleus was stained with DAPI, and images were taken by using a Leica confocal microscope.

### 4.17. Flow Cytometry

Flow cytometric measurements were done in a BD LSRFortessa™ Cell Analyzer (BD Biosciences, San Jose, CA, USA). FITC channel was used to capture the DCFDA, annexin V, and other FITC tagged markers, whereas PE-CF594 was used to capture the Mitosox and other PE tagged markers signal. A total of twenty-thousand events were acquired per sample. The data was analyzed by BD facs DivaTM and FlowJo™ (BD Biosciences, San Jose, CA, USA). The experiments were repeated three times.

### 4.18. Ethics Statement

The experiments on the animals were conducted following the Committee for the Purpose of Control and Supervision of Experiments on Animals (CPCSEA), Government of India guidelines. The protocol was approved by NIPER-Kolkata Institute Animal Ethics Committee (IAEC). The approval number for this study is IAEC/NIPER/03.

### 4.19. Statistical Analysis

Statistical data analysis was evaluated with the results obtained from the three independent experiments. Statistical analysis has been carried out in R; for the pairwise-statistical test, one-way ANOVA was used; *p* ≤ 0.05 is considered significant (* *p* ≤ 0.05, ** *p* < 0.001, ** *p* < 0.0001, and **** *p* < 0.00001) and plotting were carried out using ggplot2.

## Figures and Tables

**Table 1:** Sequence of primers used for qPCR.

Primers	Sequence (5′ to 3′)
β-actin F	AGCTTACTGCTCTGGCTCCTAGC
β-actin R	ACTCATCGTACTCCTGCTTGCT
COX-2 F	TGGTGCCTGGTCTGATGATG
COX-2 R	GTGGTAACCGCTCAGGTGTTG
iNOS2 F	CCCTCCTGATCTTGTGTTGGA
iNOS2 R	TCAACCCGAGCTCCTGGAA
MyD88 F	GTTGTGTGTGTCCGACCGT
MyD88 R	GTCAGAAACAACCACCACCATGC
TRAF 6 F	CATCTTCAGTTACCGACAGCTCAG
TRAF 6 R	TGGTCGAGAATTGTAAGGCGTAT
TRAM F	GGCCTGGACCATCTTGTTAC
TRAM R	CATGGGTATGACGGAGTTGT
NF-κB F	CCAAAGAAGGACACGACAGAATC
NF-κB R	GGCAGGCTATTGCTCATCACA
c-JUN F	GGCAGGCTATTGCTCATCACA
c-JUN R	GAAGTTGCTGAGGTTGGCCTA
FAS F	CGCTGTTTTCCCTTGCTG
FAS R	CCTTGAGTATGAACTCTTAACTGTGAG
c-FOS F	AGAGCGGGAATGGTGAAGA
c-FOS R	TCTTCCTCTTCAGGAGATAGCTG
TLR4 F	CTGGGTGAGAAAGCTGGTAA
TLR4 R	AGCCTTCCTGGATGATGTTGG
TRIF F	TGGCAAACACCTTCAAGACA
TRIF R	GCGCTTTCTTCCAGCGTA

## Data Availability

Data generated from the current study are available with corresponding authors upon reasonable request.

## Supplementary Materials

The supporting information can be downloaded at: https://www.mdpi.com/article/10.3390/ijms231911254/s1.

## Abbreviations

BGG	β-Glucogallin
COX 2	Cyclooxygenase-2
iNOS 2	Inducible Nitric Oxide Synthase 2
LPS	Lipopolysaccharide
MMP9	Matrix metallopeptidase 9
MYD88	Myeloid differentiation primary response 88
NF-KB	Nuclear factor kappa-light-chain-enhancer of activated B cells
TLR4	Toll-Like Receptor 4
TRAFs	Tumor necrosis factor receptor-associated factors
TRAM	Translocation associated Membrane Protein
TRIF	TIR-domain-containing adapter-inducing interferon-β
