# Supplementary material for: Glucogallin Attenuates the LPS-Induced Signaling in Macrophages and Protects Mice against Sepsis
Source: Int J Mol Sci. 2022 Sep 24;23(19):11254. doi: 10.3390/ijms231911254 (PMC9569803; doi:10.3390/ijms231911254)
Supplement: Supplementary file 1 [file ijms-23-11254-s001.zip › ijms-1887225-supplementary.pdf]

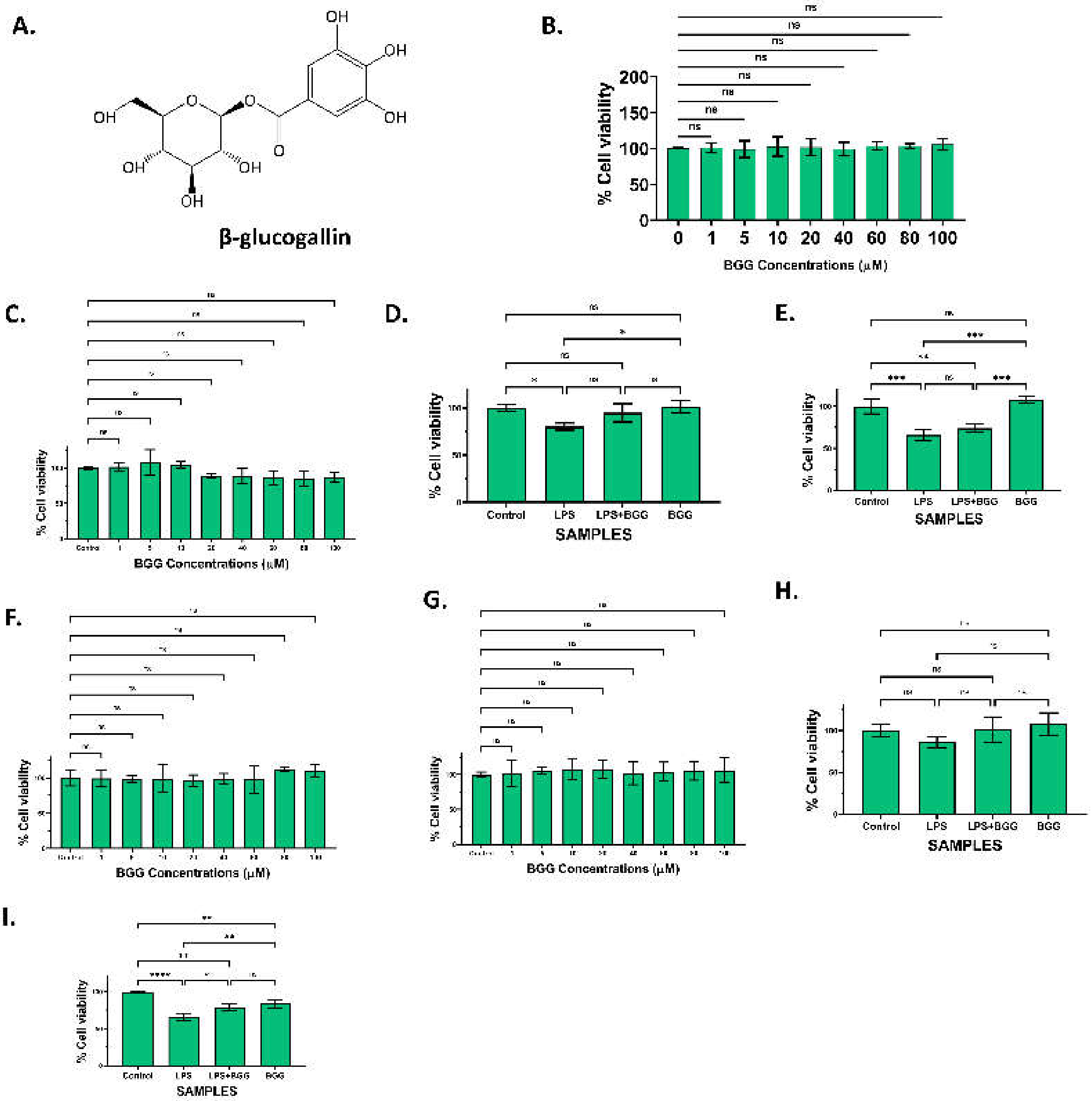


**Figure S1.** (**A**) Chemical Structure of BGG, Cell viability of various concentrations of BGG on RAW 264.7 and mice peritoneal macrophages measured with MTT, (**B**) 24 h and (**C**) 48 h on RAW 264.7 cells, (**D**) 24 h and (**E**) 48 h on mice peritoneal macropahges. Measurement of BGG (10 µM) on LPS (1µg/mL) induced cell death (**F**) 24 h, (**G**) 48 h on RAW 264.7 cells, (**H**) 24 h and (**I**) 48 h on mice peritoneal macrophages. Results are mentioned as mean ± SD (n = 5), *p*-value ≤0.05. (* $p \leq 0.05$, ** $p < 0.01$, *** $p < 0.001$, and **** $p < 0.0001$).

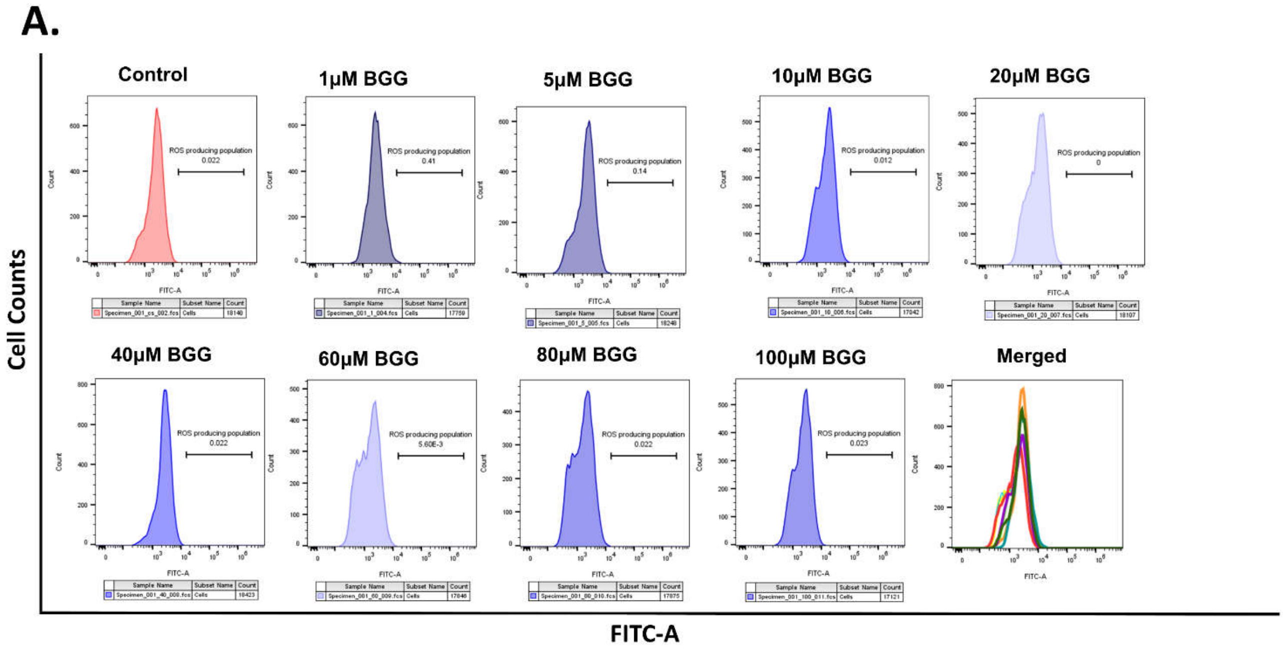


**B.**

| BGG concentrations (in μM) | Mean fluorescence intensity(MFI) |
|---|---|
| 0 (Control) | 2519 |
| 1 | 2516 |
| 5 | 2546 |
| 10 | 2253 |
| 20 | 1623 |
| 40 | 2426 |
| 60 | 1714 |
| 80 | 1720 |
| 100 | 2283 |

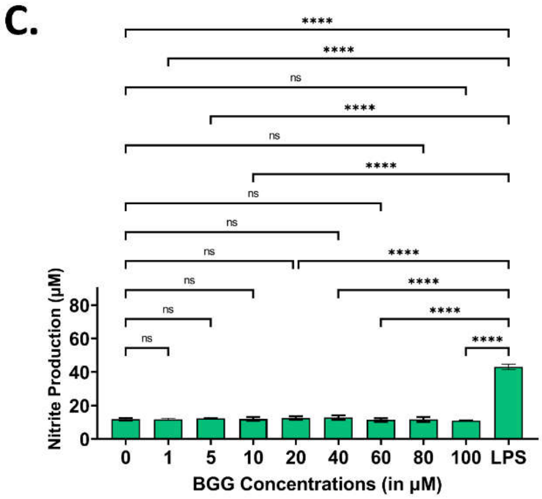


**Figure S2.** DCFDA-based total reactive oxygen species (ROS) production assay on the various concentration of BGG on RAW264.7 macrophages (**B**) and their respective mean fluorescence intensity (**C**) Extracellularly NO assay of different concentrations of BGG on RAW 264.7 macrophages. Results are mentioned as mean ± SD, (* $p \leq 0.05$, ** $p < 0.001$, *** $p <$ 1e-04, and **** $p <$ 1e-05) (n = 3 independent experiments).

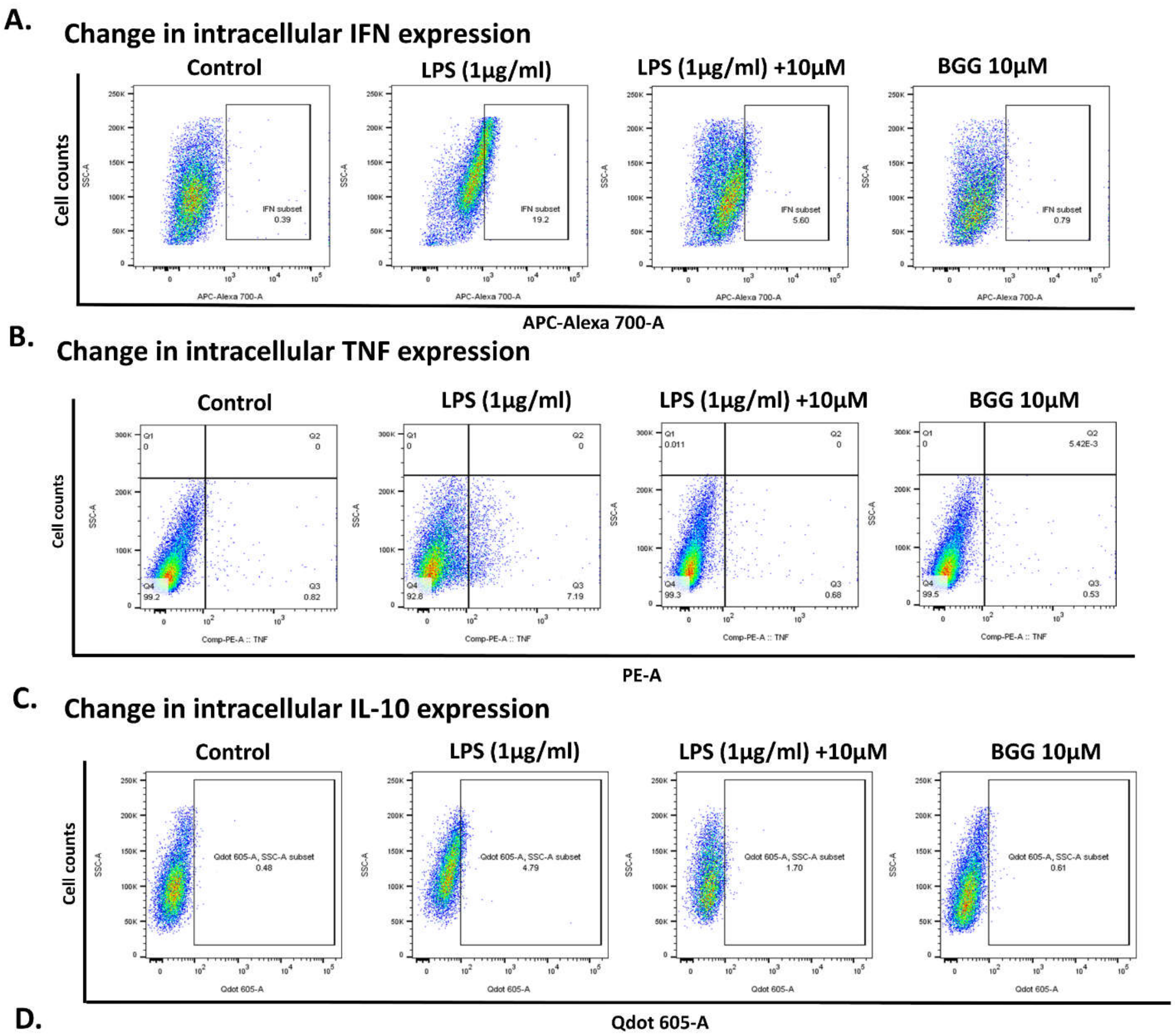


**D.**

| | TNF (%age expression) | IFN (%age expression) | IL10 (%age expression) |
|---|---|---|---|
| Control | 0.82 | 0.29 | 0.40 |
| LPS | 7.19 | 19.2 | 4.79 |
| LPS+BGG | 0.68 | 5.60 | 1.70 |
| BGG | 0.53 | 0.79 | 0.61 |

**Figure S3.** Measuring Intracellular cytokine interleukins by Flow Cytometry (**A**) Change in expression of IFN (**B**) Change in expression of IL-10 (**C**) Change in expression of TNF and (**D**) Table represents the change in expression(%age) of interleukin in percentage.

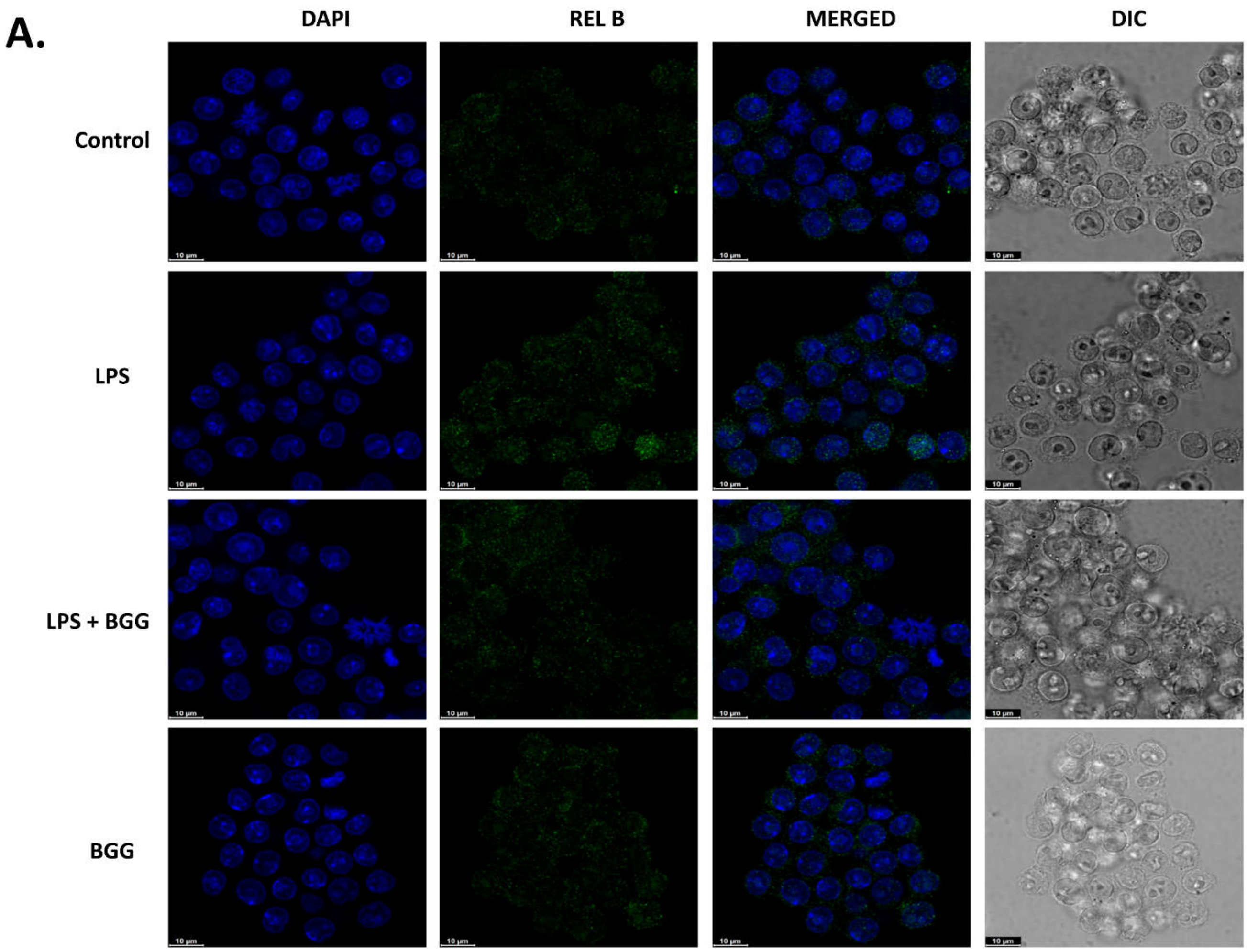


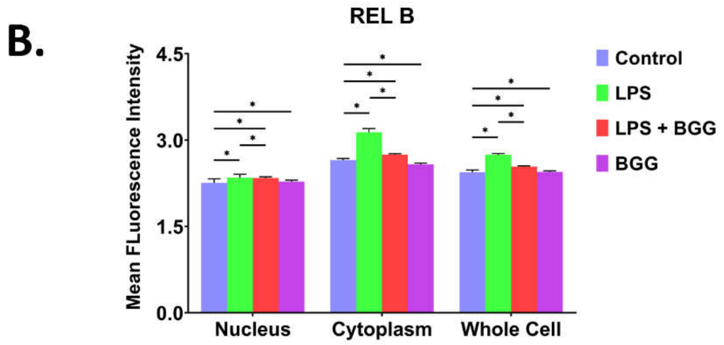


**Figure S4.** Immunofluorescence staining (**A**) To measure the translocation of RelB from the cytoplasm to the nucleus, and (**B**) Graph represents the mean fluorescence intensity of RelB in the nucleus, cytoplasm, and whole cells, (* $p \leq 0.05$.

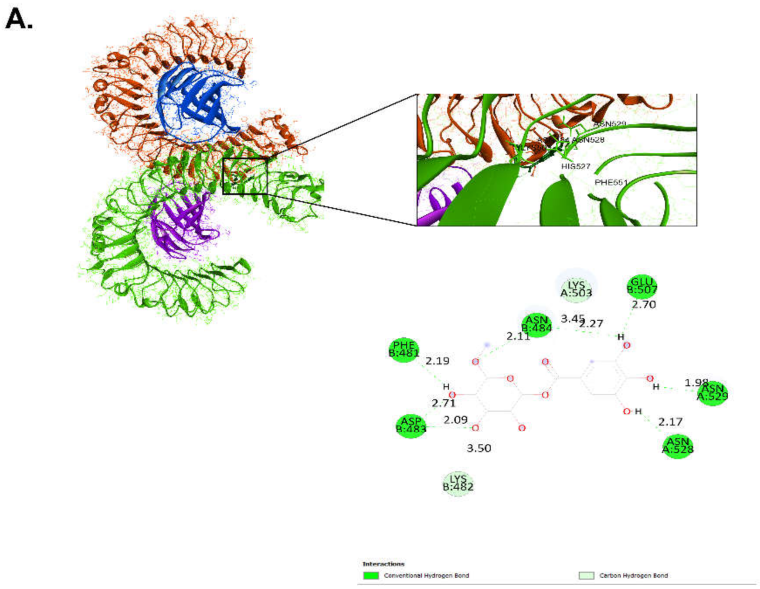


B.

| Selected Targets | PDB ID | BINDING ENERGY(kcal/mol) | INTERACTING RESIDUES |
|---|---|---|---|
| TLR4 | 3VQ2 | - 7.0 | PHE:481, ASN:484, GLU:507, ASN:529, ASN:528, LYS:503, LYS:482 |

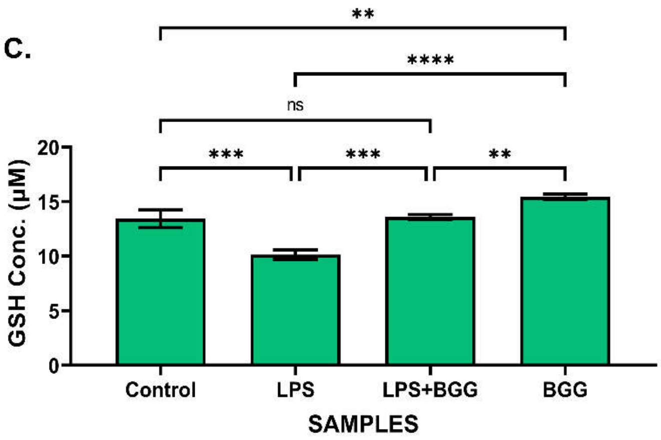


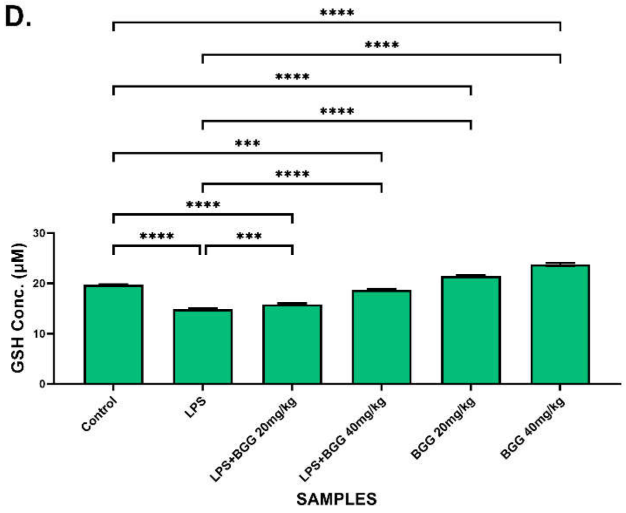


**Figure S5.** Molecular docking of BGG against (**A**) TLR4, (**B**) Table represents the PDB ID, binding energy, and interacting residues and (**C**) Represents the GSH assay with cell lysate, and (**D**) Represents the GSH assay with treated animal mice serum, (* $p \leq 0.05$, ** $p < 0.001$, *** $p <$ 1e-04, and **** $p <$ 1e-05).
